# Comparison of drug-eluting bead transarterial chemoembolization combined with apatinib versus drug-eluting bead transarterial chemoembolization for the treatment of unresectable hepatocellular carcinoma: a randomized, prospective, multicenter phase III trial

**DOI:** 10.1038/s41392-024-02012-x

**Published:** 2024-11-13

**Authors:** Xuhua Duan, Hao Li, Donglin Kuang, Pengfei Chen, Mengfan Zhang, Tengfei Li, Dechao Jiao, Yanliang Li, Xiang He, Cheng Xing, Haibo Wang, Yaoxian Liu, Limin Xie, Shixi Zhang, Qiang Zhang, Peixin Zhu, Yongchuang Chang, Jichen Xie, Jianzhuang Ren, Xinwei Han

**Affiliations:** 1 Department of Interventional Radiology, The First Affiliated Hospital of Zhengzhou University, Zhengzhou, Henan China; 2 Department of Interventional and Oncology, Dengzhou People’s Hospital, Nanyang, Henan China; 3 Department of Medical Imaging, Huaihe Hospital of Henan University, Kaifeng, Henan China; 4 Department of Interventional Radiology, Zhoukou Central Hospital, Zhoukou, Henan China; 5 Department of Interventional Radiology, Zhengzhou Central Hospital, Zhengzhou, Henan China; 6 Department of Interventional Radiology, Luohe Central Hospital, Luohe, Henan China; 7 Department of Interventional Radiology, Shangqiu First People’s Hospital, Shangqiu, Henan China; 8 Department of Infection, Shangqiu Municipal Hospital, Shangqiu, Henan China; 9 Department of Interventional Radiology, Anyang District Hospital, Anyang, Henan China; 10 Department of Interventional Radiology, General Hospital of Pingmei Shenma Group, Pingdingshan, Henan China; 11 Department of Interventional Radiology, The People’s Hospital of Anyang city, Anyang, Henan China; 12 Department of Interventional Radiology, The Fifth People’s Hospital of Puyang City, Puyang, Henan China

**Keywords:** Cancer, Cancer

## Abstract

This randomized, prospective, multicenter (12 centers in China) phase III trial (Chinese Clinical Trial Registry #ChiCTR2000041170) compared drug-eluting bead transarterial chemoembolization (DEB-TACE) combined with apatinib and DEB-TACE monotherapy for patients with unresectable hepatocellular carcinoma (uHCC). Progression-free survival (PFS) was the primary endpoint. Overall survival (OS), mRECIST-based objective response rates (ORR) and disease control rates (DCR), and treatment-related adverse events (TRAEs) were secondary endpoints. Totally 243 cases were randomized, with 122 and 121 in the DEB-TACE + apatinib and DEB-TACE groups, respectively. Cases administered DEB-TACE + apatinib displayed markedly improved median PFS (7.1 months [95%CI 6.6–8.3] vs. 5.2 months [95%CI 5.0–5.9]) and OS (23.3 months [95%CI 20.7–29.6] vs. 18.9 months [95%CI 17.9–20.1] compared with those treated with DEB-TACE (both *p* < 0.001). Additionally, patients administered DEB-TACE + apatinib had elevated ORR (56.6% vs. 38.8%) and DCR (89.3% vs. 80.2%) versus the DEB-TACE group (both *p* < 0.001). Majority of TRAEs were mild and manageable. Regarding DEB-TACE-related TRAEs, the rates of hepatic artery thinning and spasms were elevated during the second DEB-TACE in cases administered DEB-TACE + apatinib vs. DEB-TACE. The commonest apatinib-related TRAEs in the DEB-TACE + apatinib group included hypertension, hand-foot syndrome, fatigue, and diarrhea. In conclusion, DEB-TACE plus apatinib demonstrates superior PFS versus DEB-TACE monotherapy in uHCC cases, maintaining a favorable safety profile with similar occurrences of AEs.

## Introduction

Primary liver carcinoma constitutes the sixth commonest (about 865,269 incident cases in 2022, accounting for 4.3% of all malignancies) and the third deadliest (about 757,948 deaths in 2022, accounting for 7.8% of all cancer-related deaths) malignancy globally.^1^ Hepatocellular carcinoma (HCC) accounts for 70–90% of the cases.^1^ The management of HCC is multidisciplinary and encompasses surgery, systemic therapy, targeted therapy, radiotherapy, and interventional therapy.^2–4^ Unfortunately, HCC is often detected at late stages, and the tumor is unresectable, limiting the treatment options and resulting in a poor prognosis.^5,6^ Few effective therapeutic options are available for unresectable HCC (uHCC), and the options mostly include systemic treatments.^2–4^ Sill, interventional radiology can be used. Indeed, transarterial chemoembolization (TACE) is widely employed to treat uHCC with high efficacy.^4,7^ Conventional TACE (cTACE) effectively manages tumor growth by obstructing tumor blood vessels and inducing local ischemia and hypoxia. This process involves delivering a suspension of lipiodol with chemotherapeutic agents and embolic materials into HCC’s tumor-feeding artery.^8^ TACE will deprive the tumor cells of oxygen and nutrients, leading to their death.^9^ Nevertheless, cTACE-triggered hypoxia and low-glucose environment may elevate the risk of tumor angiogenesis, recurrence, and metastasis by inducing hypoxia-inducible factor-1α (HIF-1α)/vascular endothelial growth factor (VEGF) signaling.^10,11^ Therefore, suppressing VEGF expression and its associated pathways in tumor tissue may effectively reduce tumor angiogenesis after cTACE, enhancing the efficacy of TACE.^8,12^

Sorafenib, the first oral multikinase suppressor that targets the VEGF receptor (VEGF-R), RAF, and platelet-derived growth factor (PDGF) receptor, exerts antiangiogenic and direct antitumor effects, making it the standard first-line targeted drug for clinical advanced uHCC.^13^ Sorafenib monotherapy demonstrated survival benefits in the SHARP^14^ and AP^15^ phase III randomized clinical trials, where advanced HCC patients who received sorafenib exhibited prolonged survival compared to those who received placebo. Therefore, sorafenib is considered a potential candidate for combination with TACE. Kudo and co-workers conducted a study combining TACE with sorafenib, improving median progression-free survival (PFS) to 25.2 vs. 13.5 months with TACE alone in patients with uHCC.^16^ Combining cTACE with molecularly targeted agents has been shown to effectively decrease tumor angiogenesis following cTACE. Apatinib, a molecular targeted agent selectively targeting VEGF receptor-2 (VEGFR-2), displays a 10-fold higher binding affinity to VEGFR-2 tyrosine kinase versus sorafenib.^17^ In a multicenter phase II study, apatinib demonstrated efficacy and safety in front-line treatment of advanced HCC, exhibiting median overall survival (OS) times of 9.82 and 9.71 months in the 750 mg and 850 mg cohorts, respectively.^18^ A real-world study also showed apatinib was effective in patients with advanced HCC, with a tolerable safety profile.^19^ Similar to sorafenib, apatinib has shown synergistic effects with TACE in treating rabbit VX2 liver tumors as well as in HCC patients by suppressing angiogenesis within the hypoxic tumor environment induced by cTACE.^20–22^ Apatinib was proven effective in advanced HCC.^18,19,23^ Indeed, a phase II trial showed an overall response rate (ORR) of 30% and a disease control rate of 65% for apatinib.^23^

Still, cTACE has limitations, e.g., the motility of lipiodol that dilutes the chemotherapeutic drug, the inability of TACE to deliver the drug in a sustained manner, and heterogeneity in TACE techniques.^24^ An alternative to cTACE is using drug-eluting beads (DEBs) as an innovative drug delivery system in TACE. In the past decade, DEB-TACE was designed with the goal of enhancing overall treatment outcomes and mitigating the side effects associated with cTACE.^25,26^ DEBs can carry double doses of chemotherapeutic drugs compared with cTACE agents and gradually release them, thereby increasing the intratumoral drug concentration and exposure over time and effectively targeting tumor tissues.^26^ DEB-TACE extends the duration of contact between HCC cells and chemotherapeutic agents while reducing the adverse reactions of chemotherapeutic agents in the liver microcirculation, thereby potentially minimizing systematic cytotoxicity compared with cTACE.^4^ Using non-soluble microspheres in DEB-TACE improves embolic effectiveness, offering greater convenience and feasibility in clinical practice.^7^ Hence, DEB-TACE improves therapeutic efficacy by enhancing antitumor activity and establishes a standardized approach for intravascular intervention therapy in liver cancer, setting it apart from cTACE.^27^ A meta-analysis showed elevated ORR and disease control rate (DCR) and prolonged survival with DEB-TACE compared to cTACE, but could not conclude on safety due to heterogeneity in reporting among the included studies.^28^

Since both treatments are relatively new, a very small number of trials have examined DEB-TACE plus apatinib for therapeutic efficacy in HCC. Two retrospective studies confirmed the effectiveness and safety of DEB-TACE + apatinib in managing Barcelona clinic liver cancer (BCLC) stage C HCC^21^ or huge HCC.^29^ Considering the added value of DEB-TACE over cTACE, investigating the DEB-TACE + apatinib is important in HCC management. However, evidence supporting this combination therapy remains limited, and the specific benefits of DEB-TACE for HCC cases remain unknown. Therefore, this phase III trial aimed to assess DEB-TACE + apatinib vs. DEB-TACE monotherapy for efficacy and safety in patients with uHCC unsuitable for ablation procedures.

## Results

### Participant demographics

**Fig. 1:**
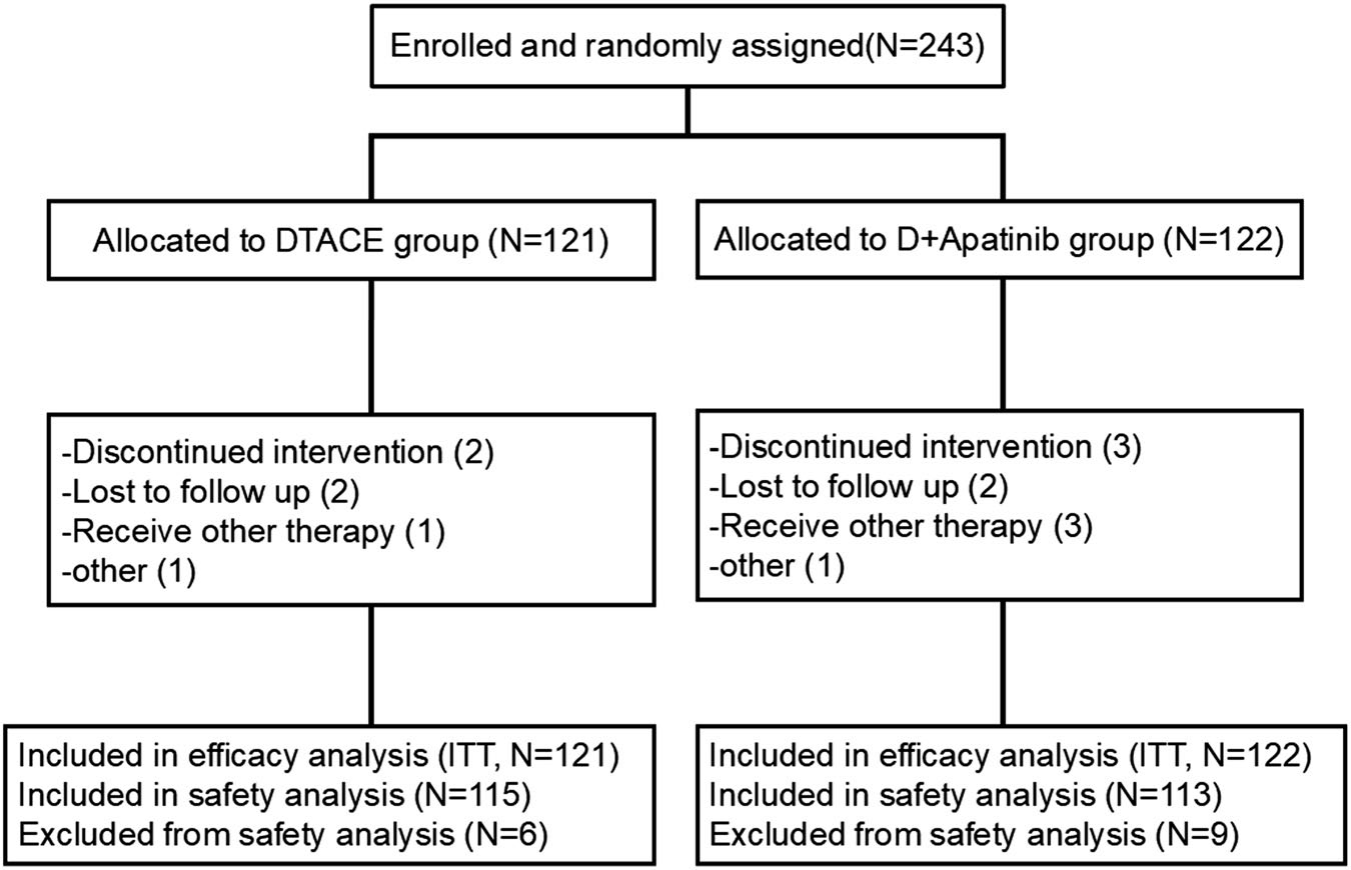
Study flowchart. Abbreviations**:** DEB-TACE, Drug-eluting bead transarterial chemoembolization; D + apatinib, DEB-TACE plus apatinib; ITT, intention-to-treat population

Between January 1, 2021 and June 30, 2022, 243 patients with uHCC were assessed for eligibility in 12 hospitals in China, enrolled, and randomized. The efficacy analysis set comprised 121 and 122 in the DEB-TACE and DEB-TACE + apatinib groups, respectively. The safety analysis set comprised 115 and 113 in the DEB-TACE and DEB-TACE + apatinib groups, respectively, according to the intention-to-treat population (Fig. 1). Both groups had comparable features at baseline (all *p* > 0.05) (Table 1).

**Table 1:** Patient Characteristics

Characteristics	DEB-TACE (*n* = 121)	DEB-TACE + apatinib (*n* = 122)	*P* value
**At Baseline**			
Sex (%)			0.216
Male	107 (88.4)	100 (82.0)	
Female	14 (11.6)	22 (18.0)	
Age (years), median (range)	58.8 ± 11.1	57.5 ± 10.2	0.354
Age group (years) (%)			0.565
<58	60 (49.6)	55 (45.1)	
≥58	61 (50.4)	67 (54.9)	
ECOG PS (%)			0.564
0	70 (57.9)	76 (62.3)	
1	51 (42.1)	46 (37.7)	
No. of lesions, *n* (%)			0.774
≤3	98 (81.0)	96 (78.7)	
>3	23 (19.0)	26 (21.3)	
Tumor distribution (%)			0.450
Bilobar	40 (33.1)	47 (38.5)	
Unilobar	81 (66.9)	75 (61.5)	
Maximum tumor diameter (cm, mean ± SD) (%)	9.3 ± 4.2	9.7 ± 4.5	0.507
<10 cm	70 (57.9)	66 (54.1)	0.646
≥10 cm	51 (42.1)	56 (45.9)	
Portal vein invasion, *n* (%)			0.697
None	51 (42.1)	58 (47.5)	
Vp1-2	52 (43.0)	48 (39.3)	
Vp3-4	18 (14.9)	16 (13.1)	
Hepatic vein invasion, *n* (%)	40 (33.1)	36 (29.5)	0.647
Extrahepatic metastasis, *n* (%)	18 (14.9)	17 (14.2)	0.834
Etiology (%)			0.363
Hepatitis B	106 (87.6)	99 (81.1)	
Hepatitis C	2 (1.7)	4 (3.3)	
Non-B Non-C	13 (10.7)	19 (15.6)	
Child-Pugh class (%)			0.838
A	101 (83.5)	104 (85.2)	
B*	20 (16.5)	18 (14.8)	
AFP (ng/mL) (%)			0.259
≤400	78 (64.5)	69 (56.6)	
>400	43 (35.5)	53 (43.4)	
BCLC stage (%)			0.376
B	40 (33.1)	48 (39.3)	
C	81 (66.9)	74 (60.7)	
**During follow-up**			
Vascular lake in DTACE (%)			0.105
No	98 (81.0)	87 (71.3)	
Yes	23 (19.0)	35 (28.7)	
No. of DTACE (%)			
1–2	42 (34.7)	31 (25.4)	0.149
≥3	79 (65.3)	91 (74.6)	
Duration of apatinib (months)			NA
≤3 months	0	38 (31.1)	
>3 months	0	84 (68.9)	
Dose reductions due to AE	0	41 (33.6)	NA
Dose pauses due to AE	0	16 (13.1)	NA

*DEB-TACE* drug-eluting beads transarterial chemoembolization, *ECOG PS* Eastern Cooperative Oncology Group Performance Status, *BCLC* Barcelona Clinic Liver Cancer, *AFP* alpha-fetoprotein. *All patients with Child-Pugh class were Child-Pugh 7

### Tumor responses

Table 2 summarizes tumor responses following the initial TACE. According to modified Response Evaluation Criteria in Solid Tumors (mRECIST) criteria, cases administered DEB-TACE + apatinib demonstrated significantly higher ORR (56.6% vs. 38.8%) and DCR (89.3% vs. 80.2%) versus the DEB-TACE group (*p* < 0.001). According to RECIST 1.1, both groups had similar responses.

**Table 2:** Best tumor response based on mRECIST and RECIST after the first DTACE between the two groups

	mRECIST, *n* (%)			RECIST 1.1, *n* (%)		
	DEB-TACE (*n* = 121)	DEB-TACE + apatinib (*n* = 122)	*P* value	DEB-TACE (*n* = 121)	DEB-TACE + apatinib (*n* = 122)	*P* value
Tumor response						
CR	9 (7.4)	14 (11.5)	0.036	0	0	0.066
PR	38 (30.4)	55 (45.1)		39 (32.2)	54 (44.3)	
SD	50 (41.3)	40 (32.8)		56 (46.3)	53 (43.4)	
PD	24 (19.8)	13 (10.7)		26 (21.5)	15 (12.3)	
ORR (CR + PR)	47 (38.8)	69 (56.6)	0.006	39 (32.3)	54 (44.3)	0.054
DCR (CR + PR + SD)	97 (80.2)	109 (89.3)	0.046	95 (78.5)	107 (87.7)	0.056

*RECIST* Response Evaluation Criteria in Solid Tumors, *mRECIST* modified RECIST, *DEB-TACE* drug-eluting beads transarterial chemoembolization, *CR* complete response, *PR* partial response, *SD* stable disease, *PD* progressive disease, *ORR* objective response rate, *DCR* disease control rate

### Survival and disease progression

On July 1, 2023 (data cutoff), median follow-up in the DEB-TACE group was 24.3 months, compared with 23.9 months in the DEB-TACE + apatinib group (*p* = 0.81). Totally 134 participants died, including 79 administered DEB-TACE (disease progression, *n* = 29; liver failure, *n* = 18; gastrointestinal hemorrhage, *n* = 9; septic shock due to spontaneous bacterial peritonitis, *n* = 7; pulmonary infection, *n* = 8; hepatic encephalopathy, *n* = 2; hepatorenal syndrome, *n* = 2; biliary tract infection, *n* = 3; pulmonary embolism, *n* = 1) and 55 administered DEB-TACE + apatinib (disease progression, *n* = 18; liver failure, *n* = 14; gastrointestinal hemorrhage, *n* = 8; septic shock due to spontaneous bacterial peritonitis, *n* = 4; pulmonary infection, *n* = 5; hepatic encephalopathy, *n* = 3; biliary tract infection, *n* = 2; cerebral hemorrhage, *n* = 1).

**Fig. 2:**
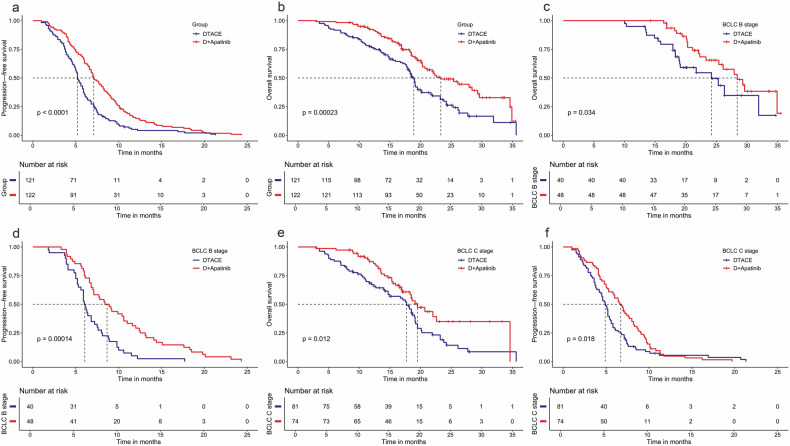
Kaplan-Meier curves for overall survival (**a**, **c**, and **e**) and progression-free survival (**b**, **d**, and **f**) estimation in both groups in all patients (**a**, **b**) and in cases with BCLC B (**c**, **d**), and BCLC C (**e**, **f**) lesions. Median (95%CI). DEB-TACE Drug-eluting bead transarterial chemoembolization, D + apatinib, DEB-TACE plus apatinib, BCLC Barcelona clinic liver cancer, *P* value for the log-rank test

The participants treated with DEB-TACE + apatinib had markedly prolonged median OS compared with those administered DEB-TACE (23.3 months [95% confidence interval (CI) 20.7–29.6] vs. 18.9 months (95%CI 17.9–20.1), *p* < 0.001), as well as starkly longer PFS (7.1 months [95%CI 6.6–8.3] vs. 5.2 months [95%CI 5.0–5.9], *p* < 0.001). Participants stratified by BCLC stage showed similar results (Fig. 2). Specifically, BCLC B cases administered DEB-TACE + apatinib exhibited markedly prolonged median OS (28.4 months [95%CI 24.2–32.7] vs. 24.2 months (95%CI 17.0–31.4), *p* = 0.034) and PFS (8.4 months [95%CI 6.7–10.1] vs. 6.0 months [95%CI 5.5–6.5], *p* < 0.001) versus the DEB-TACE group; DEB-TACE + apatinib also significantly increased median OS (19.5 months [95%CI 17.0–22.1] vs. 17.7 months [95%CI 16.1-19.3], *p* = 0.012) and PFS (6.6 months [95%CI 5.5–7.7] vs. 4.9 months [95%CI 4.5–5.3], *p* = 0.018) versus DEB-TACE in BCLC C cases. Moreover, cases administered DEB-TACE + apatinib showed higher 1-year survival rate versus those treated with DEB-TACE (86.1% vs. 71.9%, *p* < 0.05). The numbers of progression in the DEB-TACE + apatinib and DEB-TACE groups (1.93 ± 0.95 vs. 2.05 ± 0.96, *p* = 0.328) were similar in both groups, as well as the occurrence rates of lung metastases at diagnosis (14.2% vs. 14.9%, *p* = 0.834). However, as treatment progressed, the numbers of participants developing new lung metastases (9/105 vs. 20/103, *p* = 0.024) and the frequency of lateral hepatic branching artery involvement in the second or subsequent DEB-TACE (5.8% vs. 14.8%, *p* = 0.021) were notably reduced in cases administered DEB-TACE + apatinib versus DEB-TACE. Cox frailty results were hazard ratio (HR) = 0.564 (0.435–0.733) for PFS and HR = 0.526 (0.371–0.744) for OS. HRs for OS and PFS for each participating center are shown in Table S1. As shown in Table S2, the ORR was better with DEB-TACE + apatinib versus DEB-TACE in participants with hepatic vein tumor thrombosis (mRECIST, 44.4% vs. 20.0%, *P* = 0.022).

**Fig. 3:**
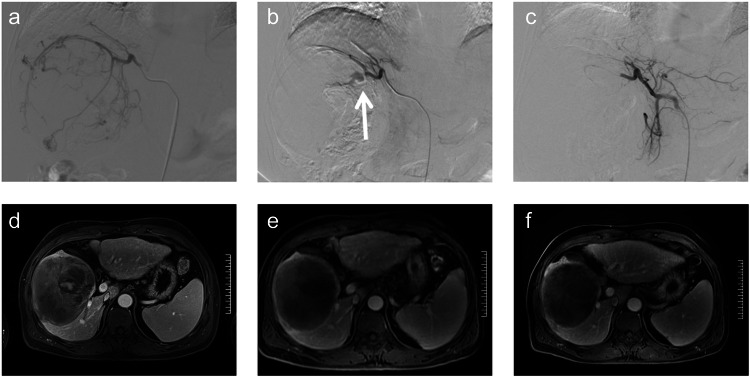
**Images in a 59-year-old man with advanced hepatocellular carcinoma. a** Hepatic angiography during the first TACE. **b** In the first DEB-TACE, CalliSpheres@ Beads with a diameter of 100-300 um loaded with 60 mg of doxorubicin were used to embolize the right hepatic artery, and the VL phenomenon occurred in the re-examination angiography (arrow). **c** PVA particles with a diameter of 350-560 μm were subsequently added for embolization until the VL phenomenon disappeared in the re-examination angiography. **d** Enhanced MRI at 4 weeks after the first DEB-TACE revealed partial contrast enhancement in the peripheral intrahepatic lesion, suggesting a partial response according to mRECIST criteria. Each small division on the scale bar represents 1 cm. **e** After 3 rounds of DEB-TACE plus apatinib, MR images at 6 months of follow-up show that the tumor had a complete response. Each small division on the scale bar represents 1 cm. **f** One-year follow-up MR images show that the tumor had a complete response. Each small division on the scale bar represents 1 cm

Multivariable analysis revealed several independent predictive factors of OS and PFS, demonstrating that DEB-TACE + apatinib showed favorable outcomes across most of the key subgroups for OS (HR = 0.55, 95%CI 0.39–0.80) and PFS (HR = 0.54, 95%CI 0.41–0.71). Notably, factors markedly correlated with OS included grouping (HR = 0.59, 95%CI 0.41–0.854), portal vein invasion Vp1-2 (HR = 2.09, 95%CI 1.01–4.35), portal vein invasion Vp3-4 (HR = 3.98, 95%CI 1.82–8.68), presence of a vascular lake (VL) in DEB-TACE (HR = 0.55, 95%CI 0.35–0.87) (Fig. 3), and ≥3 TACE sessions (HR = 0.53, 95%CI 0.36–0.79) (Table S3). For PFS, the independent factors associated were grouping (HR = 0.55, 95%CI 0.42–0.72), hepatic vein invasion (HR = 1.66, 95%CI 1.13–2.45) and ≥3 TACE sessions (HR = 0.55, 95%CI 0.41–0.75) (Table S4). Additionally, a subgroup analysis detailing factors correlated with OS and PFS in individuals administered DEB-TACE versus DEB-TACE + apatinib can be found in Figure S1 and Figure S2, respectively.

### Safety

DEB-TACE-related adverse events (AEs), e.g., fever, pain, gastrointestinal reactions, nausea, vomiting, new ascites, and liver abscess, were similar in both groups. However, during the second DEB-TACE, the rates of hepatic artery thinning (34.5% vs. 21.7%, *p* = 0.004) and spasms (16.8% vs. 9.6%, *p* = 0.035) were higher in patients administered DEB-TACE + apatinib versus those treated with DEB-TACE (Table 3). Hepatic artery spasms were significantly alleviated after administering papaverine in the second TACE. In addition, five participants in both groups had liver abscesses following DEB-TACE, four experienced relief upon abscess drainage and drainage tube removal, and one was stabilized upon anti-inflammatory therapy. There were no fatalities resulting from liver abscesses or related complications. Table 4 outlines the detailed apatinib-related AEs in cases administered DEB-TACE + apatinib. The most observed apatinib-related AEs were hypertension (43.4%), hand-foot syndrome (HFSR) (40.7%), fatigue (37.2%), and diarrhea (22.1%). No interruptions in apatinib treatment occurred, and no grade 5 AEs were reported in cases administered DEB-TACE + apatinib. Changes in liver and kidney functions are summarized in Table S5, indicating similar ALT, total bilirubin, and blood nitrogen urea in both groups. The DEB-TACE + apatinib group showed lower AST at 1 month (67.68 ± 53.98 vs. 85.02 ± 66.68 U/L, *P* = 0.027), higher albumin at 1 month (34.17 ± 10.67 vs. 28.13 ± 13.73 g/L, *P* < 0.001), and lower serum creatinine at 3 months (51.23 ± 26.59 vs. 59.94 ± 25.14 µmol/L, *P* = 0.011) vs. cases administered DEB-TACE. ALBI score and Child-Pugh class were similar in both groups (Table S6).

**Table 3:** Adverse events related to DTACE

Adverse events, *n* (%)	Any Grade			Grade 3 or 4		
	DEB-TACE (*n* = 115)	DEB-TACE + apatinib (*n* = 113)	*P* value	DEB-TACE (*n* = 115)	DEB-TACE + apatinib (*n* = 113)	*P* value
Fever	58 (50.4)	55 (48.7)	0.671	0	0	–
Pain	49 (42.6)	54 (47.8)	0.255	2 (1.7)	3 (2.7)	0.519
Gastrointestinal reaction	38 (33.0)	42 (37.2)	0.346	1 (0.9)	0	–
Nausea and vomiting	34 (29.6)	36 (31.9)	0.572	0	0	–
New ascites	13 (11.3)	15 (13.3)	0.512	3 (2.6)	2 (1.8)	0.476
Liver abscess	2 (1.7)	3 (2.7)	0.519	0	1 (0.9)	–
Hepatic artery thinning in second DTACE	25 (21.7)	39 (34.5)	0.004	0	0	–
Hepatic artery spasm in second DTACE	11 (9.6)	19 (16.8)	0.035	0	0	–

Data are numbers of events. Data in parentheses are percentages. *DEB-TACE* drug-eluting beads transarterial chemoembolization

**Table 4:** Apatinib adverse events in the DEB-TACE + apatinib group (*n* = 113)

Adverse events, n (%)	Any Grade	Grade 3	Grade 4
Hypertension	49 (43.4)	10 (8.8)	1 (0.9)
Hand foot syndrome	46 (40.7)	8 (7.1)	0 (0.0)
Fatigue	42 (37.2)	2 (1.8)	0 (0.0)
Diarrhea	25 (22.1)	1 (0.9)	0 (0.0)
Albuminuria	20 (17.7)	5 (4.4)	1 (0.9)
Gastroenteritis	18 (15.9)	0 (0.0)	0 (0.0)
Gastrointestinal hemorrhage	12 (10.6)	4 (3.5)	3 (2.7)
Thrombocytopenia	11 (9.7)	3 (2.7)	0 (0.0)
Abdominal pain	9 (8.0)	3 (2.7)	0 (0.0)
Rash	8 (7.1)	0 (0.0)	0 (0.0)
Hepatic encephalopathy	4 (3.5)	1 (0.9)	1 (0.9)

*DEB-TACE* drug-eluting beads transarterial chemoembolization. Data are numbers of events

Data in parentheses are percentages

### Follow-up after study treatments

Different treatment modalities were chosen for each participant according to the progression of their lesions. After disease progression, all participants received new systemic treatment regimens, and some underwent local treatments. In cases administered DEB-TACE + apatinib, all participants had immunotherapy or were switched to another targeted drug (donafenib or lenvatinib) combined with immunotherapy. In the DEB-TACE group, all participants were administered targeted drugs combined with immunotherapy, and none had DEB-TACE + apatinib after disease progression. In addition, 113 participants experienced local recurrence and underwent repeat DEB-TACE treatment, with two of these participants receiving additional DEB after two cTACE sessions; 39 participants developed new small lesions within the liver and underwent cTACE followed by ablation treatment; 48 participants developed lung metastases, with five of these participants undergoing microwave ablation and one undergoing cryoablation; 37 participants developed lymph node metastases, with four of these participants receiving lymph node iodine 125 seed implantation; four participants developed bone metastases, one developed spleen metastasis, and one developed brain metastasis. Treatment regimen data and overall survival are still being evaluated.

## Discussion

Previous reports have confirmed that cTACE + apatinib could achieve better clinical outcomes in patients with advanced HCC.^30^ In a multicenter retrospective cohort study, after propensity score matching, the TACE + apatinib group (with 37.3% of cases administered DEB-TACE) exhibited an ORR of 42.5%, a DCR of 84.0%, a median PFS of 7.7 months, and a median OS of 18.0 months.^20^ In another retrospective study of BCLC C HCC, where all patients opted for DEB-TACE + apatinib, ORR, DCR, and median PFS and OS were 59.1%, 79.1%, 6.3 months, and 16.9 months, respectively.^21^ Furthermore, Li and collaborators showed DEB-TACE + apatinib improved ORR (64.7% vs. 43.6%, *p* = 0.071), median PFS (19.0 vs. 10.9 months), and median OS (25.1 vs. 13.7 months) compared with DEB-TACE alone.^29^ While it appears that Li et al.^29^ reported improved PFS and OS in huge HCC compared to a single-arm study,^21^ it is crucial to note that 41.2% (14/34) of cases administered the combination regimen were BCLC stage B.^29^

In the present study, median OS and PFS were 23.3 and 7.1 months in cases administered DEB-TACE + apatinib, respectively. These results suggest improvement compared with the 18.9 and 5.2 months achieved with DEB-TACE alone. Additionally, improvements were observed in ORR (56.6% vs. 38.8%, *p* = 0.038) and DCR (89.3% vs. 80.2%, *p* = 0.004). The findings indicate that DEB-TACE + apatinib is independently associated with increased PFS and OS versus DEB-TACE alone. This association might result from apatinib’s inhibitory effect on angiogenesis due to DEB-TACE, resulting in synergistic effects of DEB-TACE and apatinib in advanced HCC.^20,21,30,31^

The progression of advanced HCC often includes extrahepatic metastasis or target lesion progression. After TACE, the target lesion is frequently supplied by extrahepatic collateral (EHC) arteries such as the superior mesenteric, physiological, right renal, and intercostal arteries. The presence of EHC arteries can lead to treatment failure and poor outcomes.^3^ Risk factors for developing EHC arteries encompass tumor size ≥ 5 cm and prior TACE sessions.^32^ Compared with DEB-TACE alone, DEB-TACE + apatinib effectively controlled EHC arteries without increasing the risk of AEs and could even reduce them. Extrahepatic metastases are most commonly found in the lungs of patients with advanced HCC, and cases with extrahepatic metastases generally have reduced survival versus those with intrahepatic metastases only.^33^ This study showed that DEB-TACE + apatinib decreased the rates of pulmonary and extrahepatic metastases compared with DEB-TACE. As a result, DEB-TACE + apatinib conferred a longer PFS than DEB-TACE. In order to achieve the most optimal benefits for the patients, tumor blood supply should be embolized as much as possible under the condition that liver function in cases administered DEB-TACE alone remains tolerable. Meanwhile, tumor diameters in the patients were large, and the tumors often had multiple vessels for blood supply. Therefore, the pursuit of thorough embolization greatly impacted liver function, and the reaction time of embolization syndrome was longer.

In the present and prior studies, DEBs for embolization during TACE have been associated with the potential formation of VLs.^34^ Previous studies have suggested that the appearance of VLs may indicate better therapeutic efficacy.^34,35^ Subgroup analysis within this study showed no significant differences in PFS and OS in cases with VLs. The presence of VLs suggests that patients undergoing TACE alone may also experience improved prognosis (Fig. 3). DEB-TACE achieved superior therapeutic effects compared to cTACE because of its ability to induce more comprehensive embolization, unlike cTACE which uses lipiodol and can only partially embolize tumor blood vessels and supply arteries, activating the hypoxia/VEGF signaling pathway.^36^

The common DEB-TACE-related AEs included postembolization syndrome, characterized by symptoms such as abdominal pain, fever, nausea, vomiting, and transient hepatic insufficiency. These effects are manageable in clinical practice, as confirmed by a multicenter, prospective study.^31^ In the DEB-TACE + apatinib group, normal hepatic artery branches and hepatic arteries were thinner and more susceptible to spasticity during the second or subsequent TACE versus initial TACE. Similar phenomena were reported in a previous study,^20^ indicating a potential association with tumor artery normalization attributed to targeted antiangiogenic drugs.^37^

In a retrospective study treating intermediate-stage HCC with mean tumor sizes of 6.5 ± 1.2 cm using 100-300-µm CalliSpheres, grade 3/4 DEB-TACE-related AEs included a 5.9% liver abscess incidence (2/34).^38^ Another study treating advanced HCC with mean tumor sizes of 7.25 ± 2.33 cm, using either 100-300- or 300-500-µm CalliSpheres, reported a liver abscess rate of 4.1% (1/24).^39^ In this study, the liver abscess rate was maintained at 2.2% (5/228) by administering antibiotics and 40 mg methylprednisolone 3 days after DEB-TACE. Furthermore, chemotherapeutics-related AEs in DEB-TACE, such as discoloration and bone marrow toxicity, were notably scarce in the present trial.

The commonest apatinib-related AEs observed here were grade 1 or 2 hypertension, diarrhea, hand-foot skin reactions, fatigue, oral ulcers, and headache. The rate of grade 3 or 4 apatinib-related AEs was 38.1% (43/113), lower than the 77% reported by the AHELP study.^18^ These AEs could be alleviated or resolved through dose reduction, temporary drug discontinuation, and symptom management. Still, the rates of AEs appear to be lower in the present study than in previous trials of apatinib.^18,23,33,40^ The reasons might lie in the recommendation of a daily apatinib dose of 500 or 250 mg instead of the 750 mg used in the AHELP study^18^ when combined with DEB-TACE. In cases administered DEB-TACE + apatinib, dose reductions accounted for 33.6%, and pauses ( < 2 weeks) due to AEs were applied in 13.1% of patients. However, AEs were mitigated following dose adjustments and/or brief interruptions. The present findings indicate that DEB-TACE and apatinib were well-tolerated, with no serious AEs.

Some caution must be emphasized. The therapeutic regimen in the current study was selected based on the Chinese liver cancer (CNLC) guidelines.^41,42^ About 60% of patients had BCLC C HCC in both groups. The guidelines propose TACE as first-line treatment in stage IIIa cases, defined as a PS score of 0-2, Child-Pugh A/B, and vascular invasion but without extrahepatic metastasis.^41,42^ Many studies, including the present study, confirmed that TACE/DEB-TACE has promising safety and efficacy in stage IIIa HCC cases.^21,43^ The above guidelines^41,42^ also recommend TACE treatment for CNLC stage IIIb liver cancer patients who are expected to benefit from controlling intrahepatic tumor growth through TACE treatment while closely monitoring the condition of extrahepatic lymph nodes. CNLC stage IIIb was defined as a PS score of 0-2, Child-Pugh A/B, vascular invasion, and extrahepatic metastasis. Among BCLC C HCC patients, 18 and 17 in the DEB-TACE and DEB-TACE + apatinib groups, respectively, were diagnosed with CNLC stage IIIb due to enlarged hilar and retroperitoneal lymph nodes according to the Chinese guideline.^41,42^ After analyzing the patient’s baseline situation in CNLC stage IIIb, the authors expected that TACE could control the growth of intrahepatic tumors, which may benefit the patients. Therefore, in the current work, individualized TACE treatment was conducted for CNLC stage IIIb cases in the control group while closely monitoring extrahepatic metastases. Once the extrahepatic metastases progressed, lymph node iodine 125 seed implantation or systemic treatment could be performed. Although benefits were observed in patients with CNLC stage IIIb HCC in this study, the population was heterogeneous, and result interpretation should be made with cautious. Therefore, further research is needed on choosing the appropriate CNLC stage IIIb patients to achieve better results under DEB-TACE + apatinib.

Despite the encouraging outcomes, several limitations existed in the current study. First, it had an inadequate sample size, potentially introducing bias into the data analysis. Secondly, after the initial PFS, patients in both groups opted for alternative local treatments, e.g., cTACE, thermal ablation, cryotherapy, iodine 125 seed implantation, or switched to other targeted drugs (donafenib and lenvatinib), and/or added programmed death 1 (PD-1) inhibitors (camrelizumab and tislelizumab). These subsequent treatments could impact between-group OS comparison. Thirdly, due to economic constraints or concerns regarding postoperative recurrence, numerous CR patients declined surgical conversion treatment. Exclusion of patients who did undergo surgical conversion treatment limited the ability to comparatively assess long-term efficacy in both groups. Fourthly, even though median PFS and OS could be determined, follow-up continues. Additionally, subsequent treatments and survival data after progression are still being collected. Lastly, disruptions in routine examinations caused by the COVID-19 pandemic during the follow-up period might have introduced bias and influenced the presented results.

Overall, DEB-TACE plus apatinib demonstrates superior PFS compared to DEB-TACE alone in uHCC patients, exhibiting an acceptable safety profile and tolerability. This combined approach of DEB-TACE with apatinib holds promise as a new and feasible therapeutic option for managing large uHCC patients.

## Materials and methods

### Participants

This phase III multicenter, randomized, open-label study was carried out in 12 hospitals in China from January 2021 to June 2022. Here, uHCC diagnosis was based on the “Diagnostic and Therapeutic Criteria for Primary Liver Cancer” guidelines in China,^42^ considering biopsy, cytology, or diagnostic imaging (dynamic computed tomography or magnetic resonance imaging). Participants with uHCC were randomly assigned 1:1 to the DEB-TACE alone (DEB-TACE) and DEB-TACE + apatinib groups through computerized central randomization using permuted blocks (sizes of four and six).

The key eligibility criteria were: 1) uHCC patients with recurrence/metastasis confirmed by histopathology or cytology, who strictly complied with the clinical diagnostic criteria of the “diagnostic and therapeutic criteria for primary liver cancer” (2017 Edition), were ineligible for palliative surgery or radiotherapy, and had ≥1 measurable lesion based on mRECIST criteria, requiring a long diameter ≥10 mm for the measurable lesion or a short diameter ≥15 mm for the enlarged lymph node); 2) after confirming the liver cancer, no treatments were performed before DEB-TACE, such as immunotherapy, liver transplantation, surgical resection, cTACE, radiofrequency/microwave/chemical ablation, argon helium knife, ultrasonic scalpel, radiation therapy, systemic chemotherapy, and targeted therapies, e.g., sorafenib, renfatinib, apatinib, and PD-1, programmed death ligand 1 (PD-L1), and cytotoxic T-lymphocyte associated protein 4 (CTLA-4) inhibitors; 3) BCLC stage B-C and non-diffuse liver cancer; 4) age of 18 to 75 years; 5) Eastern Cooperative Oncology Group (ECOG) performance status (PS) score 0-1 within 1 week pre-enrollment; 6) liver tumor accounting for <60% of total liver volume; 7) no serious complications, e.g., hypertension, coronary heart disease, and no history of mental disease or severe allergy; 8) liver function reaching Child Pugh grade A or B, and normal or post-treatment corrected renal and coagulation functions; 9) HBV DNA < 2000 IU/ml (10^4^ copies/ml); 10) negative pregnancy test in females of childbearing potential within 7 days pre-enrollment; and 11) signing of the informed consent to participate in the trial and good compliance.

Exclusion criteria were: 1) imaging examination showing that the HCC liver tumor was huge ( ≥ 60% liver volume) or tumor thrombus in the main portal vein (occupying vessel diameter ≥50%), mesenteric vein or inferior vena cava invasion, or obvious arteriovenous fistula; 2) a history of liver transplantation, surgical resection, TACE, radiofrequency/microwave/chemical ablation, argon helium knife, ultrasonic scalpel, radiotherapy, or other local treatments, or systemic chemotherapy, oral targeted liver cancer drugs (sorafenib, renfatinib, or apatinib), or immunotherapy such as PD-1/PD-L1/CTLA-4; 3) diffuse liver cancer, known cholangiocarcinoma, mixed cell carcinoma, or fibrolamellar cell carcinoma, detected previously ( < 5 years) or other concurrent uncured cancers, except for skin basal cell carcinoma and cervical carcinoma in situ that have been cured; 4) grade ≥II myocardial ischemia or myocardial infarction and poorly controlled arrhythmia (QTc interval ≥450 ms in males and ≥470 MS in females); 5) gastrointestinal bleeding within 6 months or clear gastrointestinal bleeding tendency, e.g., esophageal varices with bleeding risk, local active ulcer lesions, or fecal occult blood ≥2+ (gastroscopy required in case of fecal occult blood at 1 + ); 6) impaired coagulation function, with INR > 1.5 or prothrombin time (PT) > ULN + 4 s), displaying bleeding tendency or under thrombolytic or anticoagulant treatment; 7) central nervous system or brain metastasis, previous and current confirmed pulmonary fibrosis, interstitial pneumonia, radiation pneumonitis, drug-related pneumonia, severe lung function impairment, HIV infection, pregnant or breast-feeding women, or scheduled liver transplantation (except for patients with previous liver transplantation); 8) expected OS < 3 months; 9) creatinine clearance rate (CCr) < 2 mg/min (CCr<2 mg/min), or 10) due to various reasons, not completed treatment plan, and no follow up within 3 months.

### DEB-TACE

All participants were administered standardized DEB-TACE at each participating institution. The tumor-supplying artery was typically detected via hepatic angiography following the Seldinger puncture technique and abdominal trunk arteriography. The tumor-feeding artery was accessed using microcatheters via super-selective catheterization. CalliSpheres (Jiangsu Hengrui Pharmaceutical, China) containing 40-60 mg of doxorubicin or epirubicin (100-300 or 300-500 μm) were slowly administered by injection into the tumor-supplying artery, following previously reported techniques.^20,21^ In case of incomplete embolization, 350-560 μm polyvinyl alcohol (PVA) particles (Hangzhou Alikang Pharmaceutical Technology, Zhejiang, China) or 300-500 μm microspheres (Jiangsu Hengrui Pharmaceutical) could be additionally utilized.

Interventional radiologists with ≥10 years of experience conducted all TACE sessions at the respective participating centers. Intravenous analgesia, combining dexmedetomidine and dezocine, was administered for 48 h from TACE initiation to manage soreness during the procedure. Following DEB-TACE, the participants received 3–5 days of liver protection and symptomatic treatments to manage embolism syndrome symptoms. DEB-TACE was discontinued in case of disease progression or a condition making DEB-TACE infeasible (e.g., tumor thrombus in the main artery trunk or vascular injury), or persistent liver dysfunction, e.g., Child-Pugh score ≥9 points lasting for over 4 weeks.

### Systemic therapy

Apatinib (Jiangsu Hengrui Pharmaceutical, Co., Ltd., Shanghai, China) was orally administered to the participants in the DEB-TACE + apatinib group for the first time 3-5 days after DEB-TACE, initially at 500 mg once daily. Apatinib administration was discontinued 3 days prior to a subsequent TACE session. In cases where participants experienced grade ≥3 AEs, the apatinib dose was reduced to 250 mg once daily, suspended, or discontinued. The maximal suspension period of apatinib was 2 weeks, with no more than two suspensions allowed. If symptomatic treatment failed to alleviate the observed AEs, discontinuation of apatinib was considered. The participants tolerating AEs at 250 or 500 mg once daily continued apatinib until tumor progression, intolerance, or death.

### Assessment of treatment efficacy

Following the first TACE procedure, participants underwent routine blood analysis, liver and kidney function assessments, coagulation function evaluation, and tumor marker detection, along with enhanced magnetic resonance imaging (MRI) and/or computed tomography (CT) scans 4-6 weeks later. Two radiologists with extended experience evaluated the scans using mRECIST criteria to determine the curative effects (best response), i.e., complete response (CR), partial response (PR), stable disease (SD), or progressive disease (PD). The evaluation based on RECIST 1.1 was used for sensitivity analysis. ORR and DCR were obtained as CR + PR and CR + PR + SD, respectively. PFS was calculated from the date of first TACE to disease progression or death, and OS from the first TACE to death or last follow-up. Subsequent TACE procedures were performed if the tumor maintained arterial blood supply per enhanced MRI and/or CT with confirmed Child-Pugh classification A/B. Treatment was continued until untreatable progression, defined by meeting DEB-TACE refractoriness criteria, intolerable toxicity or consent withdrawal. During the embolization process of DEB-TACE, embolization with drug-loaded microspheres can create new spaces within the tumor mass, resulting in the accumulation of contrast agents. This imaging manifestation appears as an early arterial phase with slow disappearance, and the contrast agent can still accumulate until the venous phase, resembling a vascular lake.

### Follow-up and safety evaluations

Complications associated with DEB-TACE included fever, nausea, vomiting, abdominal pain, constipation, ascites, liver abscesses, and hepatic artery thinning and spasm during the second DEB-TACE procedure. Apatinib-related AEs, e.g., hypertension, hand-foot syndrome, fatigue, diarrhea, gastrointestinal reactions, and liver dysfunction, were managed through symptomatic treatment. The examined participants or their families were queried about AEs, survival status, and cause of death (if indicated) post-treatment through outpatient visits, WeChat, and/or mobile phone communications. AEs were documented using the National Cancer Institute Common Terminology Criteria for Adverse Events Version 4.03 (NCI-CTCAE 4.03).

After three or more consecutive standardized and refined TACE treatments, if enhanced CT/MRI conducted 1-3 months following the last procedure showed that the intrahepatic target lesion was still in a state of disease progression (PD) compared to before the first TACE treatment based on mRECIST criteria, TACE resistance was considered. In such cases, it was necessary to promptly preclude further TACE and switch to other treatments.

### Sample size estimation

The primary endpoint was PFS. Based on previous reports and clinical experiences,^40,44,45^ the estimated median PFS for DEB-TACE + apatinib was approximately 9 months, versus around 6 months for DEB-TACE. The statistical parameters set for the test were: two-sided Class I error probability (α), 0.05; beta (β), 0.2; power, 0.8. The study aimed for a 1:1 ratio in both treatment groups. The PASS software calculated that at least 117 and 116 participants in the DEB-TACE + apatinib and DEB-TACE groups were required, respectively, considering a 5-15% potential loss to follow-up. Therefore, the total sample size for both treatment groups was 233 participants.

### Statistical methods

Continuous variates were presented as mean ± standard deviation and analyzed by Student’s t-test. Categorical variates were analyzed by the chi-squared or Fisher’s exact test for comparison. Liver and kidney functions were analyzed using repeated measure analysis of variance. Kaplan-Meier curves were utilized to analyze survival outcomes, with differences assessed by the log-rank test. Factors influencing OS and PFS were evaluated through univariable and multivariable Cox proportional hazard regression analyses, and outcomes were expressed as hazard ratios with corresponding confidence intervals. The center effect was considered random due to the enrollment condition of this study (a total of 12 centers with some having a limited number of participants). Given the data type (survival data) and the need to adjust for random effects, a Cox Frailty model was employed to adjust for the center effect, and enrollment center was included as a random intercept.^46^

## Supplementary information

Supplementary Materials

## Data Availability

The data produced or assessed in the present study are included in the published article and the associated supplementary data. The data are available from the corresponding author on reasonable request.
