# Supplementary material for: Comparison of drug-eluting bead transarterial chemoembolization combined with apatinib versus drug-eluting bead transarterial chemoembolization for the treatment of unresectable hepatocellular carcinoma: a randomized, prospective, multicenter phase III trial
Source: Signal Transduct Target Ther. 2024 Nov 13;9:304. doi: 10.1038/s41392-024-02012-x (PMC11557926; doi:10.1038/s41392-024-02012-x)
Supplement: Supplementary file 1 — Supplementary Materials [file 41392_2024_2012_MOESM1_ESM.pdf]

Supplementary Materials for

Comparison of drug-eluting bead transarterial chemoembolization combined with apatinib versus drug-eluting bead transarterial chemoembolization for the treatment of unresectable hepatocellular carcinoma: a randomized, prospective, multicenter phase III trial

Xuhua Duan, Hao Li, Donglin Kuang, Pengfei Chen, Mengfan Zhang, Tengfei Li, Dechao Jiao, Yanliang Li, Xiang He, Cheng Xing, Haibo Wang, Yaoxian Liu, Limin Xie, Shixi Zhang, Qiang Zhang, Peixin Zhu, Yongchuang Chang, Jichen Xie, Jianzhuang Ren, Xinwei Han

Correspondence to: hanxinweii@163.com

This PDF file includes:

Figures. S1 to S2

Tables S1 to S6

Clinical Study Protocol

Characteristics	Patients(n%) DTACE	Patients(n%) D+Apatinib	HR (95%CI)	P value	P for interaction
**All patients**	121	122	0.53 (0.37-0.74)	<0.001	
**Sex**					0.136
Male	107 (88.4)	100 (82.0)	0.59 (0.41-0.85)	0.005	
Female	14 (11.6)	22 (18.0)	0.23 (0.07-0.72)	0.012	
**Age group (years)**					0.917
<65	60 (49.6)	55 (45.1)	0.46 (0.26-0.81)	0.007	
≥65	61 (50.4)	67 (54.9)	0.52 (0.33-0.83)	0.006	
**ECOG PS**					0.383
0	70 (57.9)	76 (62.3)	0.54 (0.34-0.86)	0.009	
1	51 (42.1)	46 (37.7)	0.47 (0.27-0.81)	0.006	
**No. of lesions**					0.85
≤3	98 (81.0)	96 (78.7)	0.52 (0.35-0.78)	0.001	
>3	23 (19.0)	26 (21.3)	0.54 (0.26-1.12)	0.098	
**Tumor distribution**					0.839
Bilobar	40 (33.1)	47 (38.5)	0.53 (0.29-0.96)	0.038	
Unilobar	81 (66.9)	75 (61.5)	0.54 (0.35-0.82)	0.004	
**maximum tumor diameter(cm)**					0.088
<10cm	70 (57.9)	66 (54.1)	0.67 (0.42-1.08)	0.101	
≥10cm	51 (42.1)	56 (45.9)	0.38 (0.23-0.64)	<0.001	
**Portal vein invasion**					0.338
None	51 (42.1)	58 (47.5)	0.56 (0.32-0.97)	0.038	
Vp1-2	52 (43.0)	48 (39.3)	0.41 (0.23-0.71)	0.001	
Vp3-4	18 (14.9)	16 (13.1)	0.75 (0.34-1.69)	0.493	
**Hepatic vein invasion**					0.013
No	81 (66.9)	86 (70.5)	0.42 (0.27-0.64)	<0.001	
Yes	40 (33.1)	36 (29.5)	1.06 (0.58-1.94)	0.839	
**Extrahepatic metastasis**					0.568
No	103 (85.1)	105 (86.1)	0.56 (0.38-0.82)	0.003	
Yes	18 (14.9)	17 (14.2)	0.40 (0.16-0.99)	0.049	
**Aetiology (%)**					0.51
Hepatitis B	106 (87.6)	99 (81.1)	0.53 (0.36-0.78)	0.001	
Hepatitis C	2 (1.7)	4 (3.3)	0 (0-Inf)	0.999	
Non-B Non-C	13 (10.7)	19 (15.6)	0.34 (0.12-0.94)	0.038	
**Child-Pugh class**					0.002
A	101 (83.5)	104 (85.2)	0.68 (0.47-0.98)	0.041	
B	20 (16.5)	18 (14.8)	0.12 (0.04-0.42)	0.001	
**AFP (ng/mL)**					0.962
≤400	78 (64.5)	69 (56.6)	0.53 (0.34-0.82)	0.004	
>400	43 (35.5)	53 (43.4)	0.48 (0.27-0.86)	0.013	
**BCLC stage**					0.561
B	68 (56.2)	74 (60.7)	0.54 (0.35-0.89)	0.014	
C	53 (43.8)	48 (39.3)	0.51 (0.3-0.88)	0.014	
**Vascular lake in DEB-TACE**					0.252
No	98 (81.0)	87 (71.3)	0.49 (0.33-0.74)	0.001	
Yes	23 (19.0)	35 (28.7)	0.75 (0.36-1.57)	0.446	
**No. of TACE(%)**					0.588
<3	42 (34.7)	31 (25.4)	0.67 (0.37-1.22)	0.195	
≥3	79 (65.3)	91 (74.6)	0.52 (0.34-0.80)	0.003	

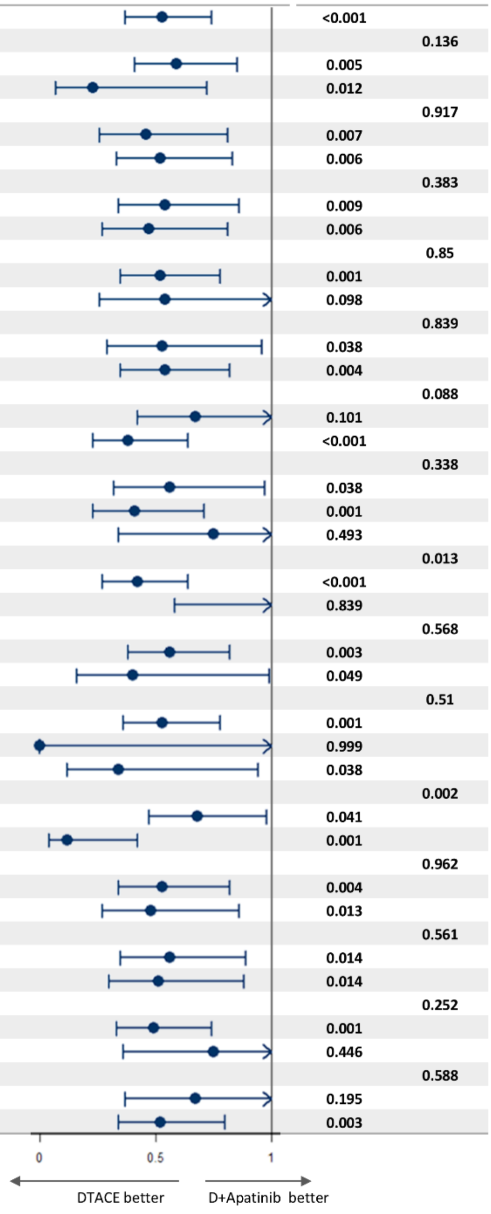

Figure. S1

Subgroup analysis of factors associated with overall survival in patients treated with DTACE versus D+Apatinib.

Abbreviations: DTACE, Drug-eluting bead transarterial chemoembolization; D + apatinib, DEB-TACE plus apatinib.

Characteristics	Patients(n%)		HR (95%CI)	P value	P for interaction
	DTACE	D+Apatinib			
**All patients**	121	122	0.57 (0.44-0.74)	<0.001	
**Sex**					0.109
Male	107 (88.4)	100 (82.0)	0.63 (0.47-0.83)	0.001	
Female	14 (11.6)	22 (18.0)	0.29 (0.14-0.63)	0.002	
**Age group (years)**					0.239
<65	60 (49.6)	55 (45.1)	0.66 (0.45-0.96)	0.032	
≥65	61 (50.4)	67 (54.9)	0.49 (0.34-0.72)	<0.001	
**ECOG PS**					0.328
0	70 (57.9)	76 (62.3)	0.49 (0.35-0.69)	<0.001	
1	51 (42.1)	46 (37.7)	0.66 (0.44-1.00)	0.051	
**No. of lesions**					0.212
≤3	98 (81.0)	96 (78.7)	0.53 (0.39-0.71)	<0.001	
>3	23 (19.0)	26 (21.3)	0.74 (0.42-1.31)	0.307	
**Tumor distribution**					0.353
Bilobar	40 (33.1)	47 (38.5)	0.67 (0.43-1.03)	0.068	
Unilobar	81 (66.9)	75 (61.5)	0.53 (0.38-0.74)	<0.001	
**maximum tumor diameter(cm)**					0.217
<10cm	70 (57.9)	66 (54.1)	0.63 (0.45-0.89)	0.009	
≥10cm	51 (42.1)	56 (45.9)	0.43 (0.29-0.65)	<0.001	
**Portal vein invasion**					0.836
None	51 (42.1)	58 (47.5)	0.52 (0.35-0.77)	0.001	
Vp1-2	52 (43.0)	48 (39.3)	0.61 (0.40-0.91)	0.016	
Vp3-4	18 (14.9)	16 (13.1)	0.69 (0.32-1.48)	0.343	
**Hepatic vein invasion**					0.589
No	81 (66.9)	86 (70.5)	0.53 (0.39-0.73)	<0.001	
Yes	40 (33.1)	36 (29.5)	0.61 (0.38-0.98)	0.042	
**Extrahepatic metastasis**					0.984
No	103 (85.1)	105 (86.1)	0.58 (0.44-0.77)	<0.001	
Yes	18 (14.9)	17 (14.2)	0.40 (0.18-0.87)	0.020	
**Aetiology (%)**					0.116
Hepatitis B	106 (87.6)	99 (81.1)	0.61 (0.46-0.81)	0.001	
Hepatitis C	2 (1.7)	4 (3.3)	0.24 (0.02-2.67)	0.244	
Non-B Non-C	13 (10.7)	19 (15.6)	0.42 (0.20-0.88)	0.021	
**Child-Pugh class**					0.540
A	101 (83.5)	104 (85.2)	0.59 (0.44-0.78)	<0.001	
B	20 (16.5)	18 (14.8)	0.48 (0.24-0.95)	0.035	
**AFP (ng/mL)**					0.450
≤400	78 (64.5)	69 (56.6)	0.52 (0.37-0.74)	<0.001	
>400	43 (35.5)	53 (43.4)	0.65 (0.43-0.98)	0.038	
**BCLC stage**					0.575
B	68 (56.2)	74 (60.7)	0.60 (0.42-0.84)	0.003	
C	53 (43.8)	48 (39.3)	0.51 (0.34-0.78)	0.002	
**Vascular lake in DEB-TACE**					0.154
No	98 (81.0)	87 (71.3)	0.51 (0.37-0.69)	<0.001	
Yes	23 (19.0)	35 (28.7)	0.79 (0.46-1.34)	0.380	
**No. of TACE(%)**					0.755
<3	42 (34.7)	31 (25.4)	0.52 (0.32-0.86)	0.011	
≥3	79 (65.3)	91 (74.6)	0.59 (0.44-0.81)	0.001	

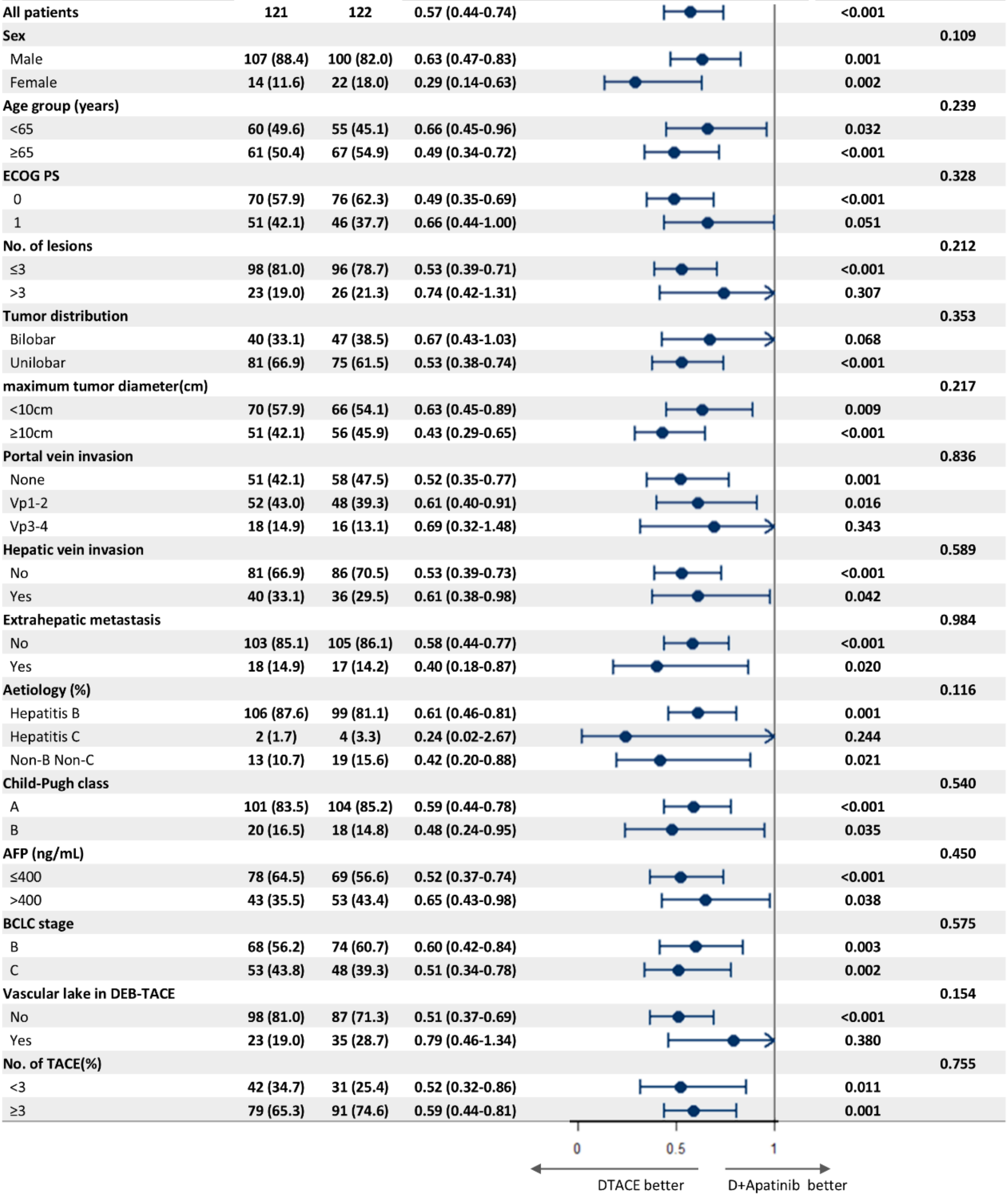

Figure S2

Subgroup analysis of factors associated with progression-free survival in patients treated with DTACE versus D+Apatinib.

Abbreviations: DTACE, Drug-eluting bead transarterial chemoembolization; D + apatinib, DEB-TACE plus apatinib.

Table S1.

Univariate Cox analysis of overall survival and progression-free survival between DEB-TACE group and DEB-TACE + apatinib group in Different Institutions.

Institutions	Group		Univariate Cox analysis			
	DTACE	DTACE + A	Overall survival		Progression-free survival	
	n(%)	n(%)	HR (95%CI)	*P* value	HR (95%CI)	*P* value
The First Affiliated Hospital of Zhengzhou University	74 (61.2)	71 (58.2)	0.54 (0.35-0.84)	0.007	0.58 (0.41-0.81)	0.001
Dengzhou People’s Hospital	8 (6.6)	6 (4.9)	1.32 (0.34-5.12)	0.689	1.24 (0.41-3.75)	0.708
Huaihe Hospital of Henan University	6 (5.0)	7 (5.7)	-	-	0.10 (0.02-0.55)	0.008
Zhoukou Central Hospital	11 (9.1)	12 (9.8)	0.51 (0.16-1.56)	0.237	0.55 (0.22-1.38)	0.202
Zhengzhou Central Hospital	3 (2.5)	4 (3.3)	0.87 (0.05-13.95)	0.919	0.50 (0.08-3.05)	0.454
Luohe Central Hospital	5 (4.1)	4 (3.3)	1.92 (0.32-11.65)	0.479	0.87 (0.21-3.51)	0.840
Shangqiu First People’s Hospital	3 (2.5)	3 (2.5)	-	-	-	-
Shangqiu Municipal Hospital	3 (2.5)	3 (2.5)	0.29 (0.03-3.42)	0.328	0.42 (0.07-2.63)	0.356

Anyang District Hospital	1 (0.8)	2 (1.6)	0.71 (0.04-11.79)	0.809	-	-
General Hospital of Pingmei Shenma Group	3 (2.5)	4 (3.3)	0.61 (0.04-9.93)	0.730	0.2 (0.02-2.05	0.177
The People's Hospital of Anyang city	3 (2.5)	5 (4.1)	0.25 (0.02-2.73)	0.253	0.34 (0.06-2.08)	0.245
The Fifth People's Hospital of Puyang City	1 (0.8)	1 (0.8)	-	-	-	-

Abbreviations: HR: hazard ratio; CI: confidence interval; DEB-TACE, drug-eluting beads transarterial chemoembolization; A: apatinib. " - " indicates that cannot be estimated.

Table S2.

Best tumor response based on mRECIST and RECIST after the first DTACE between the two groups patients with HVTT.

	mRECIST, n (%)			RECIST 1.1, n (%)		
	DEB-TACE (n=40)	DEB-TACE + apatinib (n=36)	*P* value	DEB-TACE (n=40)	DEB-TACE + apatinib group (n=36)	*P* value
Tumor response						
CR	0 (0)	1 (2.8)	0.095	0 (0)	0 (0)	0.052
PR	8 (20.0)	15 (41.7)		7 (17.5)	15 (41.7)	
SD	17 (42.5)	13 (36.1)		17 (42.5)	13 (36.1)	
PD	15 (37.5)	7 (19.4)		16 (40.0)	8 (22.2)	
ORR (CR+PR)	8 (20.0)	16 (44.4)	0.022	7 (17.5)	15 (41.7)	0.020
DCR (CR+PR+SD)	25 (62.5)	29 (80.6)	0.083	24 (60.0)	28 (77.8)	0.096

Abbreviations: RECIST, Response Evaluation Criteria in Solid Tumors; mRECIST, modified RECIST; DEB-TACE, drug-eluting beads transarterial chemoembolization; CR, complete response; PR, partial response; SD, stable disease; PD, progressive disease; ORR, objective response rate;DCR=disease control rate; HVTT: hepatic vein tumor thrombosis.

Table S3.

Univariate and multivariate Cox analysis of factors associated with overall survival.

Variables	Univariate analysis		Multivariate analysis	
	HR (95%CI)	*P* value	HR (95%CI)	*P* value
Group (DEB-TACE vs. DEB-TACE + apatinib)	0.53 (0.37-0.74)	<0.001	0.59 (0.41-0.85)	0.004
Maximum tumor diameter (≤10 vs. >10 cm)	1.69 (1.20-2.38)	0.003	1.20 (0.81-1.79)	0.364
Portal vein invasion				
None	1.00 (Ref.)		1.00 (Ref.)	
Vp1-2	2.14 (1.46-3.13)	<0.001	2.09 (1.01-4.35)	0.048
Vp3-4	5.26 (3.18-8.69)	<0.001	3.98 (1.82-8.68)	0.001
Hepatic vein invasion (No vs. Yes)	2.14 (1.49-3.07)	<0.001	1.57 (0.98-2.52)	0.058
BCLC stage (B vs. C)	2.61 (1.79-3.81)	<0.001	0.80 (0.35-1.79)	0.580
Vascular lake in DEB-TACE (No vs. Yes)	0.40 (0.26-0.62)	<0.001	0.55 (0.35-0.87)	0.012
No. of TACE (1-2 vs. ≥3)	0.42 (0.29-0.60)	<0.001	0.53 (0.36-0.79)	0.002

HR: hazard ratio; CI: confidence interval; TACE, transcatheter arterial chemoembolization; A: apatinib; BCLC, Barcelona Clinic Liver Cancer.

Table S4.

Univariate and multivariate Cox analysis of factors associated with progression-free survival.

Variables	Univariate analysis		Multivariate analysis	
	HR (95%CI)	*P* value	HR (95%CI)	*P* value
Group (DEB-TACE vs. DEB-TACE + apatinib)	0.57 (0.44-0.74)	<0.001	0.55 (0.42-0.72)	<0.001
Maximum tumor diameter (≥10 vs. <10 cm)	1.49 (1.15-1.93)	0.003	1.26 (0.92-1.72)	0.143
Portal vein invasion				
None	1.00 (Ref.)			
Vp1-2	1.37 (1.04-1.82)	0.026	1.10 (0.62-1.92)	0.751
Vp3-4	1.94 (1.31-2.89)	0.001	1.07 (0.58-1.98)	0.818
Hepatic vein invasion (No vs. Yes)	1.99 (1.50-2.63)	<0.001	1.66 (1.13-2.45)	0.010
BCLC stage (B vs. C)	1.69 (1.29-2.21)	<0.001	0.91 (0.49-1.70)	0.775
Vascular lake in DEB-TACE (No vs. Yes)	0.59 (0.43-0.80)	0.001	0.72 (0.51-1.01)	0.055
No. of TACE (1-2 vs. ≥3)	0.46 (0.35-0.62)	<0.001	0.55 (0.41-0.75)	<0.001

Duration of apatinib (≤3 months vs. >3 months)	0.62 (0.42-0.92)	0.018	NA	NA

HR: hazard ratio; CI: confidence interval; TACE: transcatheter arterial chemoembolization; A: apatinib; ECOG PS, Eastern Cooperative Oncology Group Performance Status.

Table S5.

Liver and kidney function before and after the first DEB-TACE.

	**DEB-TACE (n=121)**	**DEB-TACE + apatinib (n=122)**	**P value**
**ALT (U/L)**			
Baseline	35.36±13.96 (121)	35.45±19.93 (122)	0.966
1-month after TACE	48.82±37.78 (121)	55.44±38.79 (122)	0.181
3-month after TACE	67.97±43.27 (111)	65.30±48.71 (121)	0.662
6-month after TACE	45.62±37.77 (88)	43.75±31.99 (105)	0.711
**AST (U/L)**			
Baseline	42.30±26.57 (121)	42.85±26.72 (122)	0.873
1-month after TACE	85.02±66.68 (121)	67.68±53.98 (122)	0.027
3-month after TACE	56.08±42.22 (111)	48.52±45.19 (121)	0.193
6-month after TACE	45.62±37.77 (88)	46.86±32.82 (105)	0.809
**ALB (g/L)**			
Baseline	36.41±12.16 (121)	34.92±12.64 (122)	0.352
1-month after TACE	28.13±13.73 (121)	34.17±10.67 (122)	<0.001
3-month after TACE	32.21±7.78 (111)	32.46±7.81 (121)	0.811
6-month after TACE	31.95±8.09 (88)	29.39±12.87 (105)	0.109
**TBil (μmmol/L)**			
Baseline	23.43±14.29 (121)	21.48±13.26 (122)	0.274
1-month after TACE	22.74±16.70 (121)	24.39±17.20 (122)	0.449
3-month after TACE	22.93±14.41 (111)	23.38±15.56 (121)	0.821
6-month after TACE	25.39±18.30 (88)	23.37±13.92 (105)	0.387

CCr **(µmol/L)**			
Baseline	46.10±23.20 (121)	44.77±23.31 (122)	0.658
1-month after TACE	53.88±22.40 (121)	57.04±24.51 (122)	0.298
3-month after TACE	59.94±25.14 (111)	51.23±26.59 (121)	0.011
6-month after TACE	56.78±26.60 (88)	59.50±21.52 (105)	0.436
**BUN (mmol/L)**			
Baseline	5.14±1.21 (121)	5.17±1.28 (122)	0.856
1-month after TACE	5.08±1.96 (121)	5.34±2.36 (122)	0.366
3-month after TACE	5.49±1.78 (111)	5.47±1.76 (121)	0.952
6-month after TACE	5.67±1.62 (88)	5.55±1.88 (105)	0.663

Abbreviations: Data are shown in means±SD. (n). DTACE, Drug-eluting bead transarterial chemoembolization; D+Aptinib, DTACE conbined Apatinib; ALT: alanine aminotransferase; AST: aspartate aminotransferase; ALB: albumin; TBil: total bilirubin; CCr: Creatinine clearance rate; BUN: urea nitrogen; D+Apatinib, DTACE combined with apatinib.

Table S6.

ALBI Score and Child-Hugh Class of patients between DEB-TACE Group and DEB-TACE + apatinib Group at baesline and after the first DEB-TACE.

	**DEB-TACE Group n (%)**			**DEB-TACE + apatinib Group n (%)**			***P* value**
**ALBI Score**	**1 Score**	**2 Score**	**3 Score**	**1 Score**	**2 Score**	**3 Score**	
Baseline (121 vs. 122)	49 (40.5)	58 (47.9)	14 (11.6)	57 (46.7)	49 (40.2)	16 (13.1)	0.475
1-month (121 vs. 122)	11 (9.1)	49 (40.5)	61 (50.4)	16 (13.1)	56 (45.9)	50 (41.0)	0.290
3-month (111 vs. 121)	13 (11.7)	58 (52.3)	40 (36.0)	15 (12.4)	64 (52.9)	42 (34.7)	0.972
6-month (88 vs. 105)	10 (11.4)	45 (51.1)	23 (26.1)	18 (17.1)	57 (54.3)	30 (28.6)	0.722
**Child-Hugh Class**	**A class**	**B class**	**C class**	**A class**	**B class**	**C class**	
Baseline (121 vs. 122)	101 (83.5)	20 (16.5)	0 (0)	104 (85.2)	18 (14.8)	0 (0)	0.838
1-month (121 vs. 122)	28 (23.1)	68 (56.2)	25 (20.7)	40 (32.8)	66 (54.1)	16 (13.1)	0.128
3-month (111 vs. 121)	34 (30.6)	63 (56.8)	14 (12.6)	37 (30.6)	70 (57.9)	14 (11.6)	0.968

6-month (88 vs. 105)	37 (42.0)	41 (46.6)	10 (11.4)	36 (34.3)	52 (49.5)	17 (16.2)	0.439

Abbreviations: ALBI Score, Albumin Bilirubin Score; DEB-TACE, Drug-eluting bead transarterial chemoembolization; D+Apatinib, DTACE combined Apatinib.

Comparison of drug-eluting bead transarterial chemoembolization plus Apatinib versus DEB-TACE in the treatment of unresectable hepatocellular carcinoma: a randomized, prospective, multicenter study

Study Protocol

Sponsor: The First Affiliated Hospital of Zhengzhou University
Version No.:1.1
Version Date: 10/20/2020

Table of Contents

Protocol Signature Page 1
Protocol Synopsis...2
List of Abbreviations and Terminologies 5
Study Flow Chart 7
1. Study Background and Rationale...9
2 Study Objectives 11
2.1 Study Objectives 11
2.2 Primary Efficacy
Parameters..11
2.3 Secondary Efficacy Parameters 11
2.4 Safety Parameters...11
3 Study Design 134.1
3.1 Patient Enrollment and Randomization 13
3.1.1Patient
Enrollment...1
3
3.1.2Registration
period 13
3.1.3 Official enrollment
period 13

3.1.4 Randomization 13
3.2 Overall Design 13
3.2.1 Treatment Regimen...13
3.2.2 Efficacy Evaluation and Analysis 14
3.2.3 Follow-up of Adverse Events 15
3.2.4 Survival Follow-up 15
4 Subject Selection and Withdrawal...16
4.1 Inclusion criteria 16
4.2 Removal Criteria 17
4.3 Dropout Criteria 18
4.4 Subject Withdrawal 18
4.4.1 Withdrawal Criteria 19
4.4.2 Withdrawal Procedure 19
4.4.3 Replacement of Withdrawn Case...19
4.4.4 Follow-up of Withdrawn Cases 19
4.5 Discontinuation Criteria 20
4.6 Termination Criteria 20
4.7 Completion Criteria 20
5. Study Procedures 12
5.1 Observation Indicators 12
5.1.1 Demographic Data 12
5.1.2 Medical History 12
5.1.3 Physical Examination 12
5.1.4 Laboratory Tests 12
6 Assessment of Efficacy Evaluation 22
6.1 Primary Efficacy Parameters 22
6.2 Secondary Efficacy Parameters 22
7 Safety Assessments 24
7.1 Safety Parameters 24
7.2 Adverse Events 24
7.2.1 Definition of Adverse Events 25
7.2.2 Unexpected Adverse Events 25
7.2.3 Observation, Recording and Reporting of Adverse Events 25
7.2.4 Criteria for Judging the Severity of Adverse Events 26
7.2.5 Criteria for Judging the Relationship between Adverse Events and Study Drug 26

7.2.6 Treatment, Follow-up and Duration of Adverse Event Cases..........28
7.2.7 Judgment and Treatment of Abnormal Laboratory Test Indicators 29
7.3 Serious Adverse Events 29
7.3.1 Definition of Serious Adverse Events...29
7.3.2 Recording and Reporting Procedures for Serious Adverse Events/Pregnancies 30
8. Data Management and Statistical Analysis..23
8.1 Data Management...32
8.1.1 Recording Requirements for Case Report Forms 32
8.1.2. Database Establishment and Data Locking.......................................32
8.2 Statistical Analysis 33
8.2.1 Sample Size Determination 33
8.2.2 Statistical Analysis Database 33
8.2.3 Statistical Expression...33
8.2.4 Statistical Content...33
9. Study Management 35
9.1 Ethical Considerations and Informed Consent...................................35
9.1.1 Approval by Independent Ethics Committee....................................35
9.1.2 Informed Consent 35
9.2 Protection of Rights and Interests of Subjects 36
9.3 Study Management Institutions..36
9.4 Recording and Preservation of Study Data 36
9.5 Quality Control and Quality Assurance 37
9.6 Confidentiality...37
9.7 Case Report Forms 37

10. References...39

Protocol Signature Page

As a doctor/statistical analyst involved in the study, I have read the protocol for this study.

I have fully discussed the purpose and contents of this protocol with the Study Director.

I agree to conduct the study according to this protocol, to comply with its requirements, to abide by ethical regulations, and to conduct this clinical study under the guidance of Good Clinical Practice (GCP).

I agree that the contents of this protocol will be kept confidential and will not be disclosed to third parties, and that the contents of the protocol will only be used to conduct this study.

If I decide to withdraw from the study, I will also inform the site/Ethics Committee immediately in writing.

Signature:_________________

Date:_________________

Protocol Synopsis

Title: Comparison of drug-eluting bead transarterial chemoembolization plus Apatinib versus DEB-TACE in the treatment of unresectable hepatocellular carcinoma: a randomized, prospective, multicenter study

Study objective: To determine whether the efficacy of drug-eluting bead transarterial chemoembolization plus Apatinib is superior to that of DEB-TACE alone in the treatment of advanced primary HCC.

Study population and sample size: This study intends to enroll 233 systemic treatment-naive patients with unresectable hepatocellular carcinoma (uHCC).

Sponsor: The First Affiliated Hospital of Zhengzhou University

Principal investigator (PI) : Prof. Xinwei Han

Primary enrollment criteria

(1) Unresectable HCC patients with recurrence/metastasis confirmed by histopathology or cytology, who strictly comply with the clinical diagnostic criteria of the "diagnostic and therapeutic criteria for primary liver cancer" (2017 Edition), are unable to receive palliative surgery or radiotherapy, and have at least one measurable lesion (according to mrecist) requires that the long diameter of the measurable lesion should be greater than or equal to 10 mm or the short diameter of the enlarged lymph node should be greater than or equal to 15 mm; after confirming the liver cancer, no immunotherapy including liver transplantation, surgical resection, TACE, radiofrequency/microwave/chemical ablation, argon helium knife, ultrasonic scalpel, radiotherapy, systemic chemotherapy, oral liver cancer targeting drugs (sorafenib, renfatinib, apatinib) and PD-1 / PD-L1 / cdla-4 were not performed;

(2) Tumor stage: BCLC stage was B-C stage, non diffuse liver cancer;

(3) The age of the patients ranged from 18 to 75 years old;

(4) ECOG PS score within 1 week before enrollment: 0-1;

(5) Liver tumor accounted for less than 60% of life expectancy;

(6) No serious complications, such as hypertension, coronary heart disease and mental history, no severe allergic history;

(7) Liver function should reach child Pugh grade A or B, renal function and coagulation function should be normal or corrected after treatment;

(8) HBV DNA<2000 IU / ml (104 copies/ml);

(9) Women of childbearing age should have pregnancy test within 7 days before enrollment;

(10) The patients signed the informed consent to participate in the trial, and had good compliance.

Exclusion criteria:

(1) Imaging examination showed that HCC liver tumor was huge (≥60% liver volume),

or tumor thrombus in main portal vein (occupying vessel diameter ≥50%), invasion of mesenteric vein or inferior vena cava, or obvious arteriovenous fistula;

(2) Before participating in the study, he had received liver transplantation, surgical resection, TACE, radiofrequency / microwave / chemical ablation, argon helium knife, ultrasonic scalpel, radiotherapy and other local treatments. He had also experienced systemic chemotherapy, oral targeted liver cancer drugs (sorafenib, renfatinib, apatinib) and immunotherapy such as PD-1 / PD-L1 / cdla-4;

(3) Patients with diffuse liver cancer; known cholangiocarcinoma, mixed cell carcinoma and fibrolamellar cell carcinoma; previously (within 5 years) or at the same time suffering from other uncured malignant tumors, except for skin basal cell carcinoma and cervical carcinoma in situ that have been cured;

(4) Patients with grade II or above myocardial ischemia or myocardial infarction and arrhythmia with poor control (including QTc interval ≥450 ms in male and≥470 MS in female);

(5) In the past 6 months, patients with history of gastrointestinal bleeding or with clear gastrointestinal bleeding tendency, such as esophageal varices with bleeding risk, local active ulcer lesions, fecal occult blood ≥ + +, can not be included in the group; if fecal occult blood (+), gastroscopy is required;

(6) Patients with abnormal coagulation function (INR > 1.5 or prothrombin time (PT) > ULN + 4 seconds) had bleeding tendency or were receiving thrombolytic or anticoagulant therapy;

(7) Patients with central nervous system metastasis or known brain metastasis; patients with previous and current objective evidence of pulmonary fibrosis, interstitial pneumonia, radiation pneumonitis, drug-related pneumonia, severe lung function impairment, etc.; patients with joint HIV infection; pregnant women or breast-feeding patients; patients preparing for liver transplantation (except for patients with previous liver transplantation).

(8) The overall survival time was less than 3 months;

(9) Creatinine clearance rate (CR) was less than 2 mg / min (CR < 2 mg / min);

(10) Due to various reasons, the treatment plan could not be completed, and the patients were not followed up within three months.

Primary study endpoint: Progression-free survival (PFS)

Secondary study endpoint:

1) overall survival (OS)

2) Objective response rate (ORR)

3) Disease control rate (DCR)

4) Safety

5) Quality of Life (QoL)

Duration of this trial: Enrollment 18 months, follow-up 12 months, totally 30 months

Blind: Patients with uHCC were randomly assigned in a 1:1 ratio to either the DEB-TACE alone (DTACE) group or the DEB-TACE plus Apatinib (D + Apatinib) group through computerized central randomization using permuted blocks (size four and six).

Sample size calculation:

The primary endpoint of this study is PFS. Based on previous literature and clinical experiences , the estimated median PFS for D + Apatinib is approximately 9 months, while for DTACE, it is around 6 months. The statistical parameters set for the test are a two-sided Class I error probability (α) of 0.05, a beta (β) of 0.2, and a power of 0.8. The study aims for a 1:1 ratio in both treatment groups. The PASS software calculated that a minimum of 117 patients in the D + Apatinib group and at least 116 patients in the DTACE group would be required, considering a 5-15% potential loss rate. Therefore, the total sample size for both treatment groups would need to reach 233 patients for the study.

List of Abbreviations and Terminologies

Abbreviations and Terminologies	Explanation
ADR	Adverse drug reaction
AE	Adverse event
AFP	Alpha-fetoprotein
AKP	Alkaline phosphatase
ALT	Alanine transaminase
ANC	Neutrophil count
APTT	Activated partial thromboplastin time
AST	Aspartate aminotransferase
ALP	Alkaline phosphatase
BUN	Blood urea nitrogen
Cr	Creatinine
CR	Complete response
CRF	Case report form
CT	Computed tomography
CTC	Common toxicity criteria
Cm	Centimeter
DCR	Disease control rate
ECOG PS	Eastern Cooperative Oncology Group performance status
Fbg	Fibrinogen
GCP	Good clinical practice
Hb	Hemoglobin
HCC	hepatocellular carcinoma
MRI	Magnetic resonance imaging
PCC	Perihilar cholangiocarcinoma
PD	Disease progression
PR	Partial response
OS	Overall survival
PFS	Progression-free survival
RECIST	Response evaluation criteria in solid tumors
SAE	Serious adverse event
SD	Stable disease

TACE	Transcatheter arterial chemoembolization
TT	Thrombin time
TTP	Time to tumor progression
ULN	Upper limit of normal
URIC	Uric acid
VEGF	Vascular endothelial growth factor
VEGFR	Vascular endothelial growth factor receptor
WBC	White blood cells

Study Flow Chart

Item	Before treatment	Treatment period						
		Cycle 1	Cycle 2	Cycle 3	Cycle 4	Cycle 5	Cycle 6	Cycles thereafter
Signing the informed consent form	x							
Previous treatment history	x							
Vital signs	x	x	x	x	x	x	x	x
Physical examination	x	x	x	x	x	x	x	x
ECOG PS score	x	x	x	x	x	x	x	x
Blood pressure [a]	x	*	*	*	*	*	*	*
Blood routine [b]	x	x	x	x	x	x	x	x
Blood chemistry [b]	x	x	x	x	x	x	x	x
Urine routine [b]	x	x	x	x	x	x	x	x
Stool routine [b]	x	x	x	x	x	x	x	x
Electrocardiogram	x	x	x	x	x	x	x	x
Cardiac color ultrasound	x	This test should be supplemented when the ECG is abnormal						
Cardiac enzyme pattern	x	This test should be supplemented when deemed as necessary by the investigator						
Coagulation function	x	x	x	x	x	x	x	x
Thyroid function test	x	x	x	x	x	x	x	x
Chest radiography	x	x	x	x	x	x	x	x
Imaging examination	x	x	x	x	x	x	x	x
Tumor marker	x	x	x	x	x	x	x	x
Pregnancy test	x[c]							

Venous thrombosis of lower limbs	x	**x**	**x**	**x**	**x**	**x**	**x**	**x**

Note: **1. All observation indicators are performed on and examination time are the first day of this week or this cycle, with a window period of ±3 days;**

2. None of the observation indicators and examination time is affected by the length of drug withdrawal time. Relevant examinations (including imaging examination) are performed in each course according to the flow chart

3.(1) a: blood pressure is examined three times a week; (2) b: additional examination is performed when necessary; (3) c: during the trial as deemed necessary by the investigator according to the patient's condition and at the end of the study

1. Study Background and Rationale

Primary liver cancer is the sixth most common cancer and the fourth leading cause of cancer-related deaths worldwide; however, it remains a global health challenge, and its incidence is increasing [1]. Hepatocellular carcinoma (HCC) is the leading histological form of primary liver cancer, representing 75–85% of cases [1]. Given the tremendous heterogeneity of HCC, the patients' performance levels, extent of underlying liver cirrhosis, and tumor burden influence the choice of the treatment plan [2]. Surgical resection, ablation, and liver transplantation may provide curative potential for early-stage HCC.

Howerer, up to 50% HCC patients suitable for surgical resection will recur after a short recurrence-free interval especially patients with large (≥5 cm) or huge HCC (≥10 cm) [3,4]. In addition, as the size of a tumor increases, not only the incidence vascular invasion but also intrahepatic metastasis increases [5]. cTACE has a limited therapeutic effect on large or huge HCCs because they usually have multiple feeding arteries which include not only hepatic arterial branches but also extrahepatic collateral (EHC) arteries which led complete embolization of all tumor-feeders very difficult [6].

Currently, drug-eluting bead TACE (DEB-TACE) has been reported to be more effective, less chemotherapy-related systemic toxicity and post embolism syndrome than cTACE in clinical practice [7,8]. Recent studies demonstrated that DEB-TACE is suitable for controlling large HCC lesions and tumor size is a prognostic factor for HCC patients [7-9].

However, it is evidenced that TACE promotes tumor hypoxia, resulting in the upregulation of hypoxia inducible factor 1α (HIF-1α), which in turn activates the expressions of vascular endothelial growth factor receptor (VEGFR) as well as platelet-derived growth factor (PDGF), further promoting HCC tumor angiogenesis [10]. Apatinib, as one of the latest anti-angiogenic agents, is a small molecular tyrosine kinase inhibitor which selectively and potently suppresses VEGFR-2 and blocks VEGFR-2-regulated angiogenesis [11]. Several clinical trials have reported that the administration of apatinib is effective and well-tolerable in the treatment of various solid tumors (including advanced HCC, advanced gastric cancer, advanced non-small cell lung cancer) [12-14], and there is evidence indicating that the combination of cTACE with apatinib presented increased treatment efficacy compared with cTACE monotherapy in the treatment of intermediate and advanced HCC [15-17].

Only a few studies have revealed that DEB-TACE plus apatinib in advanced HCC patients could achieve a better clinical outcome [18, 19].There is a scarcity of studies examining the efficacy of DEB-TACE plus Apatinib in treating HCC. However, there remains limited evidence supporting the use of this combination therapy.

In summary, Apatinib combined with DEB-TACE is safe and operable;in addition, this combination is rational and demonstrates potential as a first-line treatment for advanced HCC, which requires further evaluation. Therefore, this phase III clinical trial is to compare Apatinib combined with DEB-TACE versus DEB-TACE monotherapy as the first-line treatment of advanced HCC.

2 Study Objectives

2.1 Study Objectives

To determine whether the efficacy of drug-eluting bead transarterial chemoembolization plus Apatinib is superior to that of DEB-TACE alone in the treatment of advanced primary HCC.

2.2 Primary Efficacy Parameters

Progression-free survival (PFS), defined as the time from study entry (i.e., signing the ICF) to tumor progression/recurrence of jaundice or death from any cause For subjects without tumor progression/recurrence or death, progression-free survival or recurrence-free survival will be censored at the date of the last valid tumor assessment.

2.3 Secondary Efficacy Parameters

Overall survival (OS), defined as the time from study entry (i.e., signing the ICF) to death from any cause. Subjects alive at the last contact will be censored at date of the last contact for overall survival.

Disease control rate (DCR): complete response (CR) + partial response (PR) + stable disease (SD), defined as the percentage of patients with CR, PR and SD (≥ 4 weeks) among patients who are evaluable for efficacy.

Objective response rate (ORR) ： complete response (CR) + partial response (PR) , defined as the percentage of patients with CR and PR (≥ 4 weeks) among patients who are evaluable for efficacy.

Quality of life score (EORTC QLQ-C30 V3.0 and HCC-18)

2.4 Safety Parameters

Vital signs, laboratory parameters, adverse events (AEs), and serious adverse events (SAEs).

AEs are coded according to the MedDra dictionary and each adverse event is assigned a system organ class (SOC) and preferred term. The severity of adverse events is evaluated according toNational Cancer Institute Common Terminology Criteria for Adverse Events Version 4.03 (NCI-CTCAE 4.03)

The endpoints which are used to evaluate safety are as follows:

- Incidence of AEs
- Incidence of drug-related AEs
- Incidence of death
- Incidence of AEs leading to treatment discontinuation and early withdrawal

3 Study Design

3.1 Patient Enrollment and Randomization

3.1.1 Patient Enrollment

Patient enrollment consists of two periods, registration and official enrollment.

3.1.2Registration period

If the patient with systemic treatment-naive advanced primary HCC meets all inclusion and exclusion criteria, the investigators should preliminarily introduce the background of the trial, register patient with preliminary enrollment intention, and assign a registration number.

3.1.3 Official enrollment period

If patient meets the criteria of systemic treatment-naive advanced primary HCC, the Eligibility Checklist should be completed, then the investigator should verify whether the patient meets all inclusion and exclusion criteria. The investigator will once again Introduce the trial to registered eligible patient, and the enrolling patient will formally sign the ICF.

3.1.4 Randomization

After the ICF has been signed, the Randomization Form should be completed before the patient is randomized. Patients with uHCC were randomly assigned in a 1:1 ratio to either the DEB-TACE alone (DTACE) group or the DEB-TACE plus Apatinib (D + Apatinib) group through computerized central randomization using permuted blocks (size four and six).

3.2 Overall Design

3.2.1 Treatment Regimen

Patients who meet the inclusion criteria will be randomized into the DEB-TACE+ Apatinib treatment group or DEB-TACE treatment group after having signed the informed consent form (ICF).

DEB-TACE+ Apatinib treatment:

All patients underwent standardized DEB-TACE at each participating institution. The tumor-supplying artery was typically identified through hepatic angiography following the Seldinger puncture technique and abdominal trunk arteriography. Subsequently, the tumor-feeding artery was accessed using microcatheters via super-selective catheterization. CalliSpheres (Jiangsu Hengrui Pharmaceutical Co., Ltd., Jiangsu, China) loaded with 40–60 mg of doxorubicin or epirubicin, ranging from 100–300 μm or 300–500 μm, were slowly injected into the tumor-supplying artery. In cases where embolization was incomplete, 350-560 μm polyvinyl alcohol (PVA) particles (Hangzhou Alikang Pharmaceutical Technology Co., Ltd., Zhejiang, China) or 300–500 μm microspheres (Jiangsu Hengrui Pharmaceutical Co., Ltd., Jiangsu, China) could be additionally utilized.

All medications should be recorded in detail (including drug name and dosage). Each TACE procedure was conducted by interventional radiologists with a minimum of 10 years of experience at the respective participating centers. Intravenous analgesia, a combination of dexmedetomidine and dezocine, was administered for 48 hours starting at the commencement of TACE to manage soreness during the procedure. Following DEB-TACE, patients received 3-5 days of liver protection and symptomatic treatments to manage embolism syndrome symptoms.

Apatinib dosage regimen:

The administration of Apatinib was suspended 3 days before the subsequent TACE procedure. In cases where patients experienced adverse events (AEs) of grade 3 or higher during treatment, the Apatinib dosage was reduced to 250mg once daily, suspended, or discontinued. The suspension period of Apatinib should not exceed two weeks, with no more than two suspensions allowed. If symptomatic treatment failed to alleviate adverse events, discontinuation of Apatinib was considered. Patients tolerating AEs at doses of 250mg or 500mg once daily continued Apatinib until tumor progression, patient intolerance, or patient death.

3.2.2 Efficacy Evaluation and Analysis

Following the first TACE procedure, patients underwent routine blood tests, assessments of liver and kidney functions, coagulation function, and tumor markers, along with enhanced MRI and/or CT scans 4–6 weeks later. These imaging scans were evaluated by two experienced radiologists using the modified Response Evaluation Criteria in Solid Tumors (mRECIST) to determine the best response. The curative effects were assessed based on mRECIST criteria, categorizing responses as complete response (CR), partial response (PR), stable disease (SD), or progressive disease (PD). Objective response rate (ORR) was defined as CR+PR, and disease control rate (DCR) was defined as CR+PR+SD. PFS was measured from the date of the first TACE procedure to disease progression or death. OS was measured from the first TACE to death or the last follow-up date. Subsequent TACE procedures were performed if the tumor maintained arterial blood supply as per enhanced MRI and/or CT imaging and confirmed Child–Pugh classification of A or B. Treatment was continued until untreatable progression, defined by meeting DEB-TACE fractiousness criteria, experiencing unacceptable toxicity, or withdrawal of consent.

If there is radiologically confirmed progressive disease (PD), the subject will be discontinued from the trial and will enter the follow-up period. No other anticancer therapy is allowed until PD.

For patients withdraw due to intolerable toxicity, if efficacy evaluation is not performed within 4 weeks before withdrawal, efficacy evaluation should be performed once at withdrawal, followed by imaging examination at the same frequency, i.e., every two cycles until disease progression or other anti-tumor treatment. Imaging evidence of PD of such patients should be obtained.

3.2.3 Follow-up of Adverse Events

Adverse events that are ongoing at the time of discontinuation of Apatinib should be followed up and subject to a final evaluation. Adverse events are followed up to 28 days after discontinuation.

3.2.4 Survival Follow-up

Survival (date of death and cause of death) and post study treatment information (including treatment received) will be collected by telephone questioning the subject, his/her family or a local physician every 2 month following the 28-day safety follow-up until the endpoint of death or until the subject is lost to follow-up or the investigator terminates the study. Each survival follow-up should be recorded in detail in the follow-up form.

4 Subject Selection and Withdrawal

4.1 Inclusion criteria:

Primary enrollment criteria

(1) Unresectable HCC patients with recurrence/metastasis confirmed by histopathology or cytology, who strictly comply with the clinical diagnostic criteria of the "diagnostic and therapeutic criteria for primary liver cancer" (2017 Edition), are unable to receive palliative surgery or radiotherapy, and have at least one measurable lesion (according to mrecist) requires that the long diameter of the measurable lesion should be greater than or equal to 10 mm or the short diameter of the enlarged lymph node should be greater than or equal to 15 mm; after confirming the liver cancer, no immunotherapy including liver transplantation, surgical resection, TACE, radiofrequency/microwave/chemical ablation, argon helium knife, ultrasonic scalpel, radiotherapy, systemic chemotherapy, oral liver cancer targeting drugs (sorafenib, renfatinib, apatinib) and PD-1 / PD-L1 / cdla-4 were not performed;

(2) Tumor stage: BCLC stage was B-C stage, non diffuse liver cancer;

(3) The age of the patients ranged from 18 to 75 years old;

(4) ECOG PS score within 1 week before enrollment: 0-1;

(5) Liver tumor accounted for less than 60% of life expectancy;

(6) No serious complications, such as hypertension, coronary heart disease and mental history, no severe allergic history;

(7) Liver function should reach child Pugh grade A or B, renal function and coagulation function should be normal or corrected after treatment;

(8) HBV DNA<2000 IU / ml (104 copies/ml);

(9) Women of childbearing age should have pregnancy test within 7 days before enrollment;

(10) The patients signed the informed consent to participate in the trial, and had good compliance.

Exclusion criteria:

(1) Imaging examination showed that HCC liver tumor was huge (≥60% liver volume), or tumor thrombus in main portal vein (occupying vessel diameter ≥50%), invasion of mesenteric vein or inferior vena cava, or obvious arteriovenous fistula;

(2) Before participating in the study, he had received liver transplantation, surgical resection, TACE, radiofrequency / microwave / chemical ablation, argon helium knife, ultrasonic scalpel,

radiotherapy and other local treatments. He had also experienced systemic chemotherapy, oral targeted liver cancer drugs (sorafenib, renfatinib, apatinib) and immunotherapy such as PD-1 / PD-L1 / cdla-4;

(3) Patients with diffuse liver cancer; known cholangiocarcinoma, mixed cell carcinoma and fibrolamellar cell carcinoma; previously (within 5 years) or at the same time suffering from other uncured malignant tumors, except for skin basal cell carcinoma and cervical carcinoma in situ that have been cured;

(4) Patients with grade II or above myocardial ischemia or myocardial infarction and arrhythmia with poor control (including QTc interval ≥450 ms in male and≥470 MS in female);

(5) In the past 6 months, patients with history of gastrointestinal bleeding or with clear gastrointestinal bleeding tendency, such as esophageal varices with bleeding risk, local active ulcer lesions, fecal occult blood ≥ + +, can not be included in the group; if fecal occult blood (+), gastroscopy is required;

(6) Patients with abnormal coagulation function (INR > 1.5 or prothrombin time (PT) > ULN + 4 seconds) had bleeding tendency or were receiving thrombolytic or anticoagulant therapy;

(7) Patients with central nervous system metastasis or known brain metastasis; patients with previous and current objective evidence of pulmonary fibrosis, interstitial pneumonia, radiation pneumonitis, drug-related pneumonia, severe lung function impairment, etc.; patients with joint HIV infection; pregnant women or breast-feeding patients; patients preparing for liver transplantation (except for patients with previous liver transplantation).

(8) The overall survival time was less than 3 months;

(9) Creatinine clearance rate (CR) was less than 2 mg / min (CR < 2 mg / min);

(10) Due to various reasons, the treatment plan could not be completed, and the patients were not followed up within three months.

4.2 Removal Criteria

1. Not meet the inclusion criteria, and meet the exclusion criteria;
2. Modern traditional Chinese medicine preparations and immunomodulators approved by NMPA for the treatment of hepatocellular carcinoma are used concomitantly, affecting the efficacy judgment;
3. Radiation therapy or other local therapy is performed during the study, affecting the efficacy judgment;

4. Insufficient data, affecting the judgment of efficacy and safety;

5. Failure to use drugs according to the dose, method and course of treatment specified in this protocol, affecting the judgment of drug efficacy

4.3 Dropout Criteria

1) Failure to complete treatment according to the trial protocol due to grade 3/4 adverse events in spite of dose adjustment;

2) Occurrence of unexpected and unacceptable adverse drug reactions.

3) Patients with delayed medication for > 2 weeks;

4) The subject or his/her legally acceptable representative requests to withdraw from the study;

5) Subjects lost to follow-up;

Handling of drop-out cases:

Definition of dropout: All the subjects who fill in the informed consent form and pass the screening to enter the randomized trial, no matter when they withdraw from the trial and for what reason, as long as they do not complete the observation period specified in the protocol, are all the dropout cases. The subjects who discontinue the drug after the symptoms disappear in less than a course of treatment will not be considered as dropout cases.

Treatment of dropout cases: ① When a subject drops out, the investigator should call at the subject's house, make an appointment, call, letter and other means to contact the subject as far as possible to ask the reason and record the evaluation items that can be completed when the last medication is taken. ② If the subject withdraws from the trial due to allergic reaction, adverse reaction and ineffective treatment, the investigator shall take corresponding therapeutic measures according to the actual situation of the subject. ③ The investigator should fill in the record of the main reasons for discontinuing the trial. ④ For all the patients who have been included and numbered, no matter whether they drop out or not, the observation medical records shall be recorded and retained for archiving and intention analysis (ITT). All the dropped out cases shall be summarized and statistically analyzed.

4.4 Subject Withdrawal

4.4.1 Withdrawal Criteria

1 Inability to receive treatment according to the study protocol;

2 Inability to receive treatment according to the study protocol;

3 Subject requests withdrawal;

4 Subject requests withdrawal;

5 The patient is inappropriate for continuation of therapy as judged by the investigator.

4.4.2 Withdrawal Procedure

For patients who withdraw from the study, the investigator must ask about the reason for withdrawal and whether any adverse events have occurred. The investigator should visit and assess patients who withdraw from the study, if possible. The reason for withdrawal and the date (date of the last dose) must be recorded in the case report form (CRF).

Upon withdrawal from the trial, if there are new or worsening CTC grade 3 or 4 laboratory values, the patients must receive further examinations, and the results should be recorded in the corresponding parts of the CRF until the laboratory values recover to CTC grade 1 or 2, unless the examination value cannot be improved due to the disease itself. The investigator must record in the CRF and medical record for these cases.

All study-related toxicities and SAEs present at the time of study termination must be followed until resolution, unless, in the opinion of the investigator, the condition is not likely to resolve because of the patient's disease itself.

After a patient discontinues study treatment, the investigator must follow all existing or new AEs that occur within 30 days after the last dose of study drug. All new AEs and SAEs that occurred during this time period are reported and followed until resolution of the adverse event as described above.

4.4.3 Replacement of Withdrawn Cases:

Patients who prematurely withdraw from the study will not be replaced.

4.4.4 Follow-up of Withdrawn Cases

Patients who discontinue study treatment for reasons other than progression (except withdrawal of consent, lost to follow-up) should continue to undergo objective tumor assessment every 8 weeks to collect information on disease progression. After documentation of disease progression, the investigator must contact the patient, patient's family, or the patient's current physician by telephone at least every 12 weeks to collect long-term follow-up information for survival.

4.5 Discontinuation Criteria

1) Grade 3/4 adverse events occurred during the study;

2) Pregnancy during the study.

Note: The subjects who discontinue the trial should actively receive dose adjustment with the study drug and symptomatic treatment. After comprehensive decision is made by the subjects and the investigators, the drugs can be continued according to the original protocol under the premise of discontinuation for ≤ 4 weeks. Subsequent follow-up is performed until dropout, termination or completion criteria are met.

4.6 Termination Criteria

1) Medical or ethical reasons affecting the continuation of the study;

2) The investigator judges that it is necessary to withdraw from the study from the best interests of the subjects;

3) The investigator does not comply with the approved protocol or relevant regulations to conduct this study.

4.7 Completion Criteria

1) Progressive disease (PD);

2) Death;

5. Study Procedures

5.1 Observation Indicators

5.1.1 Demographic Data

Date of birth, gender, ethnicity, marital status, height, weight, occupation, blood donation, smoking and alcohol consumption.

5.1.2 Medical History

History of present illness and treatment, other potential concomitant diseases or risk factors and treatment, significant past medical history and allergy history.

5.1.3 Physical Examination

A complete physical examination including vital signs (temperature, heart rate, respiration, blood pressure) is required.

5.1.4 Laboratory Tests

The laboratory test results obtained within 14 days before enrollment are acceptable, mainly including the following contents:

(1) Tumor markers:

Alpha-fetoprotein (AFP); Alpha-fetoprotein heteroplasmy (AFP-L3); PIVKA

(2) Blood routine

Red blood cell count (RBC), hemoglobin (Hb), white blood cell count (WBC) with differential, platelet count (PLT) and neutrophil percentage (NEU%);

(3) Urine routine

Abnormal or not, specify if abnormal.

(4) Fecal routine + occult blood

(5) Liver and kidney function as indicated by blood chemistry

Alanine transaminase (ALT), aspartate aminotransferase (AST), alkaline phosphatase (ALP), γ-glutamyltransferase (γ-GT), albumin (Alb), urea (BUN), blood creatine (Cre), serum creatinine (Cr), total bilirubin (TBIL), indirect bilirubin (DBIL), blood glucose, potassium lactate (K +), sodium (Na +), chloride (Cl +), calcium (Ca2 +), magnesium (Mg2 +).

(6) ECG, coagulation function, thyroid function.

(7) Urine pregnancy test (women of childbearing potential need to obtain negative pregnancy test results before enrollment. A pregnancy test is not required for women who are postmenopausal,

menstruating at the time of examination, or who have had contraceptive operation, but the reason for not doing so should be recorded)

6 Assessment of Efficacy Evaluation

6.1 Primary Efficacy Parameters

- Progression-free survival (PFS)

PFS is defined as the time from the date of randomization to the date of any documented tumor progression or death due to any cause. It is assessed according to RECIST 1.1 criteria, and the analysis of this indicator includes tumor evaluation results during study treatment and follow-up period. If several indicators of a patient can all be determined as PD, the first indicator should be used for PFS analysis; recurrence, appearance of new lesions or death is considered to have reached the study endpoint, and patients receiving other systemic anti-tumor treatment or anti-tumor treatment targeted to the observed target lesions are also considered to have tumor progression.

6.2 Secondary Efficacy Parameters

- Overall survival (OS): defined as the time from the date of receiving the study treatment regimen until death due to any cause.
- Disease control rate (DCR)

Refers to the percentage of the number of subjects whose best response is CR, PR or SD from the start of the study treatment regimen to the time when the subject withdraws due to disease progression out of the total number of analysis data set.

Method of evaluation: Objective tumor response is assessed using the modified Response Evaluation Criteria in Solid Tumors (mRECIST 1.1 criteria). Subjects must have measurable tumor lesions at baseline. Efficacy evaluation criteria are classified into complete response (CR), partial response (PR), stable disease (SD) and progressive disease (PD) according to RECIST 1.1 criteria. Subjects who are initially assessed as CR or PR should be confirmed 3 weeks later (the next protocol-specified efficacy assessment).

- Objective response rate (ORR)

Refers to the percentage of the number of subjects whose best response is CR or PR from the start of the study treatment regimen to the time when the subject withdraws due to disease progression out of the total number of analysis data set.

Method of evaluation: Objective tumor response is assessed using the modified Response Evaluation Criteria in Solid Tumors (mRECIST 1.1 criteria). Subjects must have measurable tumor lesions at baseline. Efficacy evaluation criteria are classified into complete response (CR), partial response (PR), stable disease (SD) and progressive disease (PD) according to RECIST 1.1 criteria. Subjects who are initially assessed as CR or PR should be confirmed 3 weeks later (the next protocol-specified efficacy assessment).

- Number of patients with adverse reactions (safety evaluation)
- Quality of life score (QoL)

Evaluation method: Observe the changes of relevant clinical symptoms and objective examination results of cancer patients before and after treatment for scoring. Timing of evaluation: before treatment, at the end of the second and third cycles of treatment. Thereafter, at the end of every two cycles, according to the content of quality of life scale, assessment record, and the requirements in the appendix, the scoring results of each domain of the scale are recorded in the CRF.

7 Safety Assessments

The clinical safety is evaluated by spontaneous reporting by patients or by direct observation by physicians or by asking patients about adverse events by non-inductive means. At the same time, blood is drawn for testing at each visit point to observe the changes in relevant indicators. Physical examination results, vital signs, adverse events and laboratory abnormalities of patients will be summarized. All adverse events should be reported and graded according to the National Cancer Institute Common Terminology Criteria for Adverse Events Version 4.03 (NCI-CTCAE 4.03).

7.1 Safety Parameters

Complete examination: height, weight, ECOG score, heart rate, blood pressure, body temperature and respiratory rate;

Blood routine: white blood cell, red blood cell, hemoglobin, platelet, neutrophil count.

Liver function: alanine transaminase (ALT), aspartate aminotransferase (AST), alkaline phosphatase (ALP), total bilirubin (TBIL), direct bilirubin (DBIL), albumin (ALB).

Renal function: blood urea nitrogen (BUN), creatinine (Cr).

Coagulation function, cardiac function

7.2 Adverse Events

Subjects must be closely monitored for adverse events. Adverse events should be assessed according to their seriousness, severity and relationship with the study drug.

The investigator is responsible for assessing the relationship of all adverse events to the study drug.

7.2.1 Definition of Adverse Events

An adverse event is any unforeseen medical condition or worsening of a pre-existing medical condition that occurs during or after administration of a medicinal product, whether or not considered related to the study drug. Unforeseen medical conditions may be symptoms (e.g. nausea, chest pain), signs (e.g. tachycardia, hepatomegaly) or abnormal findings (e.g. laboratory tests, ECG). In clinical studies, an adverse event can be an unforeseen adverse medical condition that occurs at any time from signing of informed consent form, including the screening period, even if no study treatment has been received.

Adverse events occurring in humans, whether or not related to the drug, include the following;

(1) Adverse events occurred during the use of the drug by professionals;

(2) Adverse events caused by overdose (whether intentional or unintentional);

(3) Adverse events caused by drugs of abuse;

(4) Adverse events caused by drug withdrawal;

(5) Adverse events that may occur only because of the patient's participation in the study (e.g., adverse events or serious adverse events due to discontinuation of antihypertensive drugs during the washout period) must be reported as adverse events even if they are unrelated to the study drug.

7.2.2 Unexpected Adverse Events

An unexpected adverse event is any adverse drug reaction whose characteristics or severity are not consistent with those described in the package insert of the marketed product. Supplementation of important information on the characteristics or severity of known and listed adverse events is also a part of the report of unexpected adverse events. For example, events that are more special or more serious than those described in the Investigator's Brochure should be considered "unexpected". Specific example: acute renal failure that has been indicated as an adverse event, followed by interstitial nephritis.

7.2.3 Observation, Recording and Reporting of Adverse Events

All adverse events occurring after the subject signs the informed consent form must be completely recorded in the subject's case report form. Records must be supported by source documents. Each event should be described in detail, including the start and stop date, severity, relationship with the investigational product, actions taken, and outcome of the event.

7.2.3.1 Method of Identifying Adverse Events

At each follow-up visit, adverse events can be identified through the following methods:

- Information voluntarily provided by the patient or caregiver;
- At each follow-up visit, the patient is asked an open, non-inductive question: How do you feel? Have you had any (other) medical problems since your last follow-up?
- Abnormalities are observed by the investigator, other healthcare professionals and family.

7.2.3.2 Time to Collect Adverse Events

Non-serious adverse events are recorded in this study from the time the patient gives informed consent to the 30-day follow-up period after the patient withdraws from treatment.

7.2.3.3 Collection of Adverse Event Data

All adverse events should be recorded in the CRF. The description of the adverse event includes the start and stop time, whether it is a serious adverse event, the measures taken (such as changes in the study treatment, other treatment and subsequent examinations) and the outcome. The investigator should perform the causality assessment (the relationship with the study treatment). For adverse events, CTC grading should be performed and the changes should be recorded in the corresponding CRF.

7.2.4 Criteria for Judging the Severity of Adverse Events

The following grading criteria should be used: The severity of adverse events should be recorded according to the National Cancer Institute Common Terminology Criteria for Adverse Events Version 4.03 (NCI-CTCAE 4.03).

7.2.5 Criteria for Judging the Relationship between Adverse Events and Study Drug

Criteria for judging the relationship between adverse events and study drug

According to the Provisions for Adverse Drug Reaction Reporting and Monitoring of China, the relevance evaluation is divided into four levels as not related, possibly related, probably related and related according to five criteria for adverse reaction/event analysis.

Five criteria for adverse reaction/event analysis

1. Is there a reasonable temporal relationship between medication and the occurrence of adverse reactions/events?

2. Does the reaction belong to the known type of adverse reaction of the drug?

3. Does the reaction disappear or be alleviated after discontinuation or dose reduction?

4. Does the same reaction/event recur after re-administration of the suspected drug?

5. Can the reaction/event be explained by the role of concomitant medication, patient's disease progression, and the impact of other treatments?

If it is determined to be definitely related, probably related and possibly related, it should be considered as adverse reaction caused by the drug, and whether it is a serious adverse event should be considered according to the severity.

An evaluation of the relationship of an adverse event to study drug administration is a clinical decision based on a comprehensive consideration of all available information at the completion of the CRF.

● Situations evaluated as "not related" include:

1. There is a clear alternative explanation (e.g., traumatic bleeding at the surgical site); or

2. Unreasonable (e.g., the subject was hit by a car, but there was no indication that drug-induced disorientation led to the event: or cancer that occurs only a few days after the start of drug administration)

● An evaluation of "related" indicates that there is reason to believe that the adverse event is related to the use of the study drug.

● Factors to be considered when evaluating the relationship between adverse events and the study drug include:

1. Temporal sequence with drug administration: This adverse reaction occurs after administration.

2. The event disappears after drug withdrawal (stopping stimulation) and recurs after repeated administration (repeated stimulation); the clinical course of the suspected event is analyzed to fully consider the patient's response after drug withdrawal (stopping stimulation) or the patient's response after re-administration (repeated stimulation).

3. Underlying diseases, concomitant diseases and intermittent diseases: evaluation should be performed at each report according to the natural course of related diseases, disease treatment process and all other diseases that the patient may have;

4. Concomitant medication or treatment: other drugs taken or other treatments received by the patient should be examined to determine whether one of them may cause the adverse event;

5. Known adverse reaction categories of this drug: refer to clinical data and preclinical data;

6. Pharmacology and pharmacokinetics of study drugs: The pharmacokinetic characteristics (absorption, distribution, metabolism and excretion) of study drugs should be combined with the individual pharmacodynamic response of each patient.

7. For adverse events occurring in multiple evaluation periods/cycles, their correlation evaluation results with the study drug shall be consistent.

Table Determination of Relationship between Adverse Events and Study Drug

	Definitely related	Probably related	Possibly related	Unlikely related	Definitely unrelated

Reasonable temporal sequence to administration of study drug	+	+	+	+	−
Known drug reaction type	+	+	+	−	−
Reactions abate or disappear after drug withdrawal	+	+	±	±	−
Reactions recur after re-administration	+	?	?	?	−
Unexplained by subject's disease	+	+	−	±	−

Note: + positive - negative ± possible? Unknown Definitely, probably, or possibly are counted as adverse drug reactions.

In this study, abnormalities in laboratory test indicators are not reported as adverse events if they are not clinically significant as judged by the investigator. In case of unexplained abnormal laboratory test results, the test should be immediately repeated and followed up until it return to the normal range and/or enough evidence are obtained to explain the abnormalities, and clear explanation should be recorded in the case report form.

7.2.6 Treatment, Follow-up and Duration of Adverse Event Cases

The patient is to be followed up closely after the initial report of the adverse event by the investigator. All adverse events occurring during the study, i.e., the patient has completed the study or completed the treatment, should be followed up until resolution or until the patient's condition is stable, unless it is impossible for the adverse event to alleviate due to the patient's condition according to the investigator's judgment, or the patient is lost to follow-up.

7.2.7 Judgment and Treatment of Abnormal Laboratory Test Indicators

Abnormalities in laboratory test values are first compared by the investigator with baseline and then judged for the clinically significance; abnormal laboratory test values judged as clinically significant and with change from baseline are reported as adverse events and followed up until they return to normal or baseline levels; abnormal laboratory test values without clinical significance or with clinical significance but no change from baseline are not reported as adverse

events. All abnormal laboratory test values/vital signs should be accurately recorded in the relevant CRF.

7.3 Serious Adverse Events

7.3.1 Definition of Serious Adverse Events

An SAE is defined as an AE occurring at any dose of study drug, comparator, or placebo during any period of the study (i.e., screening period, treatment period, follow-up period) that meets one or more of the following criteria:

·Results in death

·Immediately life-threatening

·Requires hospitalization or prolongation of existing hospitalization

·Results in persistent or significant disability/incapacity

·Congenital anomaly/birth defect

·Important medical events that may injure the patient or require medical treatment to prevent the occurrence of the listed conditions

Any event or hospitalization definitively caused by disease progression should not be reported as a serious adverse event. Death definitively caused by disease progression must be recorded in the CRF and reported to the monitor at the next visit, rather than reported as a serious adverse event.

Life-threatening: The term "life-threatening" is defined as "serious" and refers to an adverse event in which the subject is at risk of death at the time of the event, it does not refer to an adverse event that hypothetically might have caused death if it is more severe.

Hospitalization: Any adverse event that results in hospitalization or prolonged hospitalization is considered serious, unless it meets one of the following exceptions:

·Stay in the hospital for no more than 12 hours;

·Admission is preplanned (i.e., surgery or elective surgery that has been scheduled before starting this study);

·Admission is not associated with an adverse event (e.g., hospitalization for convalescence purposes)

·Disability, which means that someone is severely impaired in their ability to engage in daily life.

7.3.2 Recording and Reporting Procedures for Serious Adverse Events/Pregnancies

Procedures for reporting serious adverse events are as follows:

1. The investigator reports the serious adverse event to the hospital ethics committee within 24 hours;

2. The principal investigator reports to the central ethics committee within 24 hours of receiving the SAE report;

3. All investigators report to the ethics committee of each hospital as soon as possible after receiving the SAE report.

For fatal or life-threatening events, fax the copies of reports from the hospital, such as any relevant diagnostic test results/reports, including pathology reports, discharge summaries, autopsy reports, post-mortem relevant histopathological results and other documents when requested and available.

The investigator must use all necessary therapeutic measures to handle the SAE. Any medications needed to handle the SAE must be recorded in the Concomitant Medication column of the CRF for that patient.

Adverse events should be followed up until the last day of the study (including the follow-up period and withdrawal period of the study), until the study staff believe that the patient's condition has been stable, or until 30 days after the last administration of the study drug, whichever is latter.

If a serious adverse event occurs to a subject during the trial, no matter it is related to the study drug or not, the investigator should immediately take appropriate treatment measures to ensure the safety of the subjects, timely report to the clinical trial director of the site, report to the contact person (Xuhua Duan) of the sponsor (the First Affiliated Hospital of Zhengzhou University) within 24 hours after awareness, and report to Division of Study Supervision, Department of Registration, Ethics Committee, Division of Medical Care and other study institutions in this study within 24 hours in accordance with GCP regulations. After being informed of the SAE, the investigator must timely fill in the SAE form and record the occurrence time, severity, duration, actions taken and outcome of the SAE.

For all serious adverse events, the investigator is responsible for following up and providing information to relevant personnel and departments according to the reporting time limit specified above. This information should be more detailed than that recorded on the Adverse Event Report

Form. In general, this should include a detailed description of the adverse event to allow a complete medical assessment of the event and independent judgment of its possible cause. In addition to this, information on other possible causes, such as concomitant medications and diseases, must be provided. In the event of death, if any autopsy findings become available, an autopsy report must be submitted to the relevant personnel and departments as soon as possible.

For the serious adverse events related to the study drug, the sponsor should bear the cost of rescue and treatment for the patients and provide corresponding economic compensation.

8. Data Management and Statistical Analysis

8.1 Data Management

8.1.1 Recording Requirements for Case Report Forms

All cases, no matter the cases complying with the trial protocol or drop-out cases, shall be carefully filled in the case report form on the basis of complete and accurate writing of original records according to the provisions of this protocol. The principal investigator of each clinical study site shall be responsible for the authenticity of the trial data of the site.

The original laboratory test sheet must be complete and pasted on the original record. The laboratory test data or description recorded in the CRF should be checked with the original test report in the original records, and the monitor should be responsible for this.

For the laboratory test items with data beyond the clinically acceptable range, no matter before and after treatment, the investigator should verify them. The circumstances caused by non-study diseases or non-permitted concomitant diseases should be reexamined in time. The subjects with confirmed abnormalities should not be included before treatment and should be followed up to normal after treatment.

See the original records and case report form for the instructions for filling in the form.

8.1.2. Database Establishment

Establishment of database: The statistical unit undertaking this study is responsible for establishing the database and entering all the data. The data manager constructs the database according to the study protocol and CRF, sets the logic check for data validity, and opens the system access for the investigator and monitor, so as to check the data and answer questions.Randomly select several case report forms and the data in the database for manual comparison to ensure that the data in the database are consistent with the results in the original CRF. After finalizing the data blind verification report and statistical plan, the data will be locked and statistical analysis will be performed according to the statistical analysis plan.

8.2 Statistical Analysis

8.2.1 Sample Size Determination

The primary endpoint of this study is PFS. Based on previous literature and clinical experiences, the estimated median PFS for D + Apatinib is approximately 9 months, while for

DTACE, it is around 6 months. The statistical parameters set for the test are a two-sided Class I error probability (α) of 0.05, a beta (β) of 0.2, and a power of 0.8. The study aims for a 1:1 ratio in both treatment groups. The PASS software calculated that a minimum of 117 patients in the D + Apatinib group and at least 116 patients in the DTACE group would be required, considering a 5-15% potential loss rate. Therefore, the total sample size for both treatment groups would need to reach 233 patients for the study.

8.2.2 Statistical Analysis Database

Safety Set (SS): Actual data with at least one treatment and safety indicator recorded. Missing safety values should not be carried forward; some excluded cases that can be evaluated are included, such as those with age exceeding the inclusion criteria, but excluding those who use prohibited drugs, which makes safety judgment impossible. The incidence of adverse reactions takes the number of cases in the safety set as the denominator.

8.2.3 Statistical Expression

Qualitative indicators are described by frequency table, percentage or constituent ratio; quantitative indicators are described by mean, standard deviation, or maximum, minimum and median.

The report is mainly expressed in tables. The form is self-explanatory, that is, it has table title, table notes and cases.

Graphical and tabular representations of results, especially repeated measurement data, are used where necessary to increase readability.

8.2.4 Statistical Content

Safety analysis

The incidence of adverse events and adverse reactions is calculated.

If necessary, the frequency and count of adverse reactions can be listed by system, and the percentage can be calculated.

Detailed listing of cases of each adverse event.

Detailed listing of cases of each adverse reaction.

Number and conversion rate of patients with laboratory indicators and ECG turning "from normal to abnormal" or showing "abnormal aggravation" after the trial.

Cases of abnormal laboratory parameters, electrocardiograms, physical examinations, and clinical interpretations are listed.

Efficacy analysis

Imaging statistics, tumor marker indicators.

Baseline and demographic characteristics

Baseline data include: demographic characteristics, baseline tumor characteristics, medical history, concomitant medications, vital signs, etc. For measurement data, mean, standard deviation, median, minimum and maximum are used for description; for enumeration data, frequency and percentage are used for description.Comparison of mean values between multiple groups using one-way analysis of variance; homogeneity test of variance is performed before analysis of variance.

9. Study Management

9.1 Ethical Considerations and Informed Consent

9.1.1 Approval by Independent Ethics Committee

Before initiating the trial, the investigator/study institution should obtain the written approval comments from the Ethics Committee on the trial protocol, informed consent form, subject recruitment procedures and other written materials to be provided to the subjects. During the trial, if there is any new amendment to the trial protocol, informed consent form, etc., written approval from the Independent Ethics Committee should be obtained again.

9.1.2 Informed Consent

The investigator or his/her designated representative will be responsible for explaining study background, trial protocol and benefits and risks of participation in the trial to each subject, the subject's legally acceptable representative or witness, and obtain written informed consent form signed by the subject or his/her legally acceptable representative and study physician before the subject is included in the trial (before screening examination).

The text of the final informed consent form should contain the following contents: trial objective, trial procedures, obligations of the subjects, predictable benefits, risks and inconveniences from participating in the trial; treatment and appropriate insurance compensation available to the subjects in case of trial-related damage; access to trial data and confidentiality of subject information.

The informed consent form should be provided with the written approval from the Ethics Committee and in a language that is readable to the subject. The informed consent form should be signed and personally dated by the subject or by the subject’s legally acceptable representative and by the investigator or his/her representative who conducted the informed consent process. An original copy of the informed consent form is kept by the investigator and the subject respectively. If important new information involving the study is found, the informed consent form must be revised in writing and submitted to the ethics committee for approval before obtaining the consent of the subject again.

9.2 Protection of Rights and Interests of Subjects

The Ethics Committee and Informed Consent Form are the main organizations and measures to protect the rights and interests of subjects. Before the start of the clinical trial, the trial protocol can only be implemented after being reviewed and approved by the Ethics Committee of the unit responsible for the study and signed the approval comments. During the clinical trial, any amendment to the trial protocol should be approved by the Ethics Committee before implementation.

The clinical investigator must inform the subject that participation in the clinical trial is voluntary, that he/she is entitled to withdraw from the trial at any stage of the trial without discrimination or retaliation, that his/her medical treatment and rights and interests are not affected, and that he/she can continue to receive other treatment methods. The subjects must be informed that the personal data of participating in the trial and during the trial are confidential. The subjects shall also be informed of the nature of clinical trial, trial objective, expected possible benefits and possible risks and inconveniences, other alternative treatment options and the rights and obligations conforming to the provisions of Declaration of Helsinki of the subjects, so as to give the subjects sufficient time to consider whether they are willing to participate in the trial and sign the ICF.

9.3 Study Management Institutions

This study is planned to be conducted in the study institution of the First Affiliated Hospital of Zhengzhou University, and the principal investigator is Professor Xinwei Han.

9.4 Recording and Preservation of Study Data

According to the principles of GCP, the investigator should keep all the detailed original documents of the subjects, and record the contents related to the trial process, medication, laboratory test data, safety data and efficacy evaluation in the case report form. The recorded data should be complete, timely and clear. Case report forms, source documents, medical records, etc. should be clear and detailed and easily identified by the personnel participating in this clinical trial.

Case Report Forms (CRFs) are uniformly printed with Carbon-free three copies. After each CRF is completed, the first copy is used for statistical entry, the second copy is kept by the sponsor and the third copy is kept by the investigator.

The trial data shall be kept for 5 years after the completion of trial. However, these data shall be retained for a longer time if required by the current regulations or agreements with the sponsor. The sponsor will inform the investigator in writing when these data are no longer required to be retained.

9.5 Quality Control and Quality Assurance

In order to ensure the quality of the trial, the sponsor and multi-center investigators jointly discuss and develop the clinical study plan before the formal trial. Conduct GCP training for relevant study personnel participating in the trial.

According to GCP guidelines, necessary steps should be taken during the design and implementation stage of the study to ensure that the data collected are accurate, consistent, complete and credible. All the observed results and abnormal findings in clinical trials shall be timely and carefully verified and recorded to ensure the reliability of data. The instruments, equipment, reagents and standards used for various test items in the clinical trial shall have strict quality standards and ensure that they work in the normal state.

The investigator completes the information required by the protocol in the case report form (CRF). The responsible person verifies whether the information is complete and accurate, and instructs the site staff to make necessary corrections and supplements.

9.6 Confidentiality

All records regarding the subject's identity will be kept confidential and will not be made publicly available to the extent permitted by applicable laws and/or regulations.Only the subject number and initials will be recorded in the case report form. If the results of the study are published, the patient's personal identity will remain confidential. The investigator will maintain a list to facilitate reconciliation of the patient's records.

9.7 Case Report Forms

The investigator must ensure that the case report forms are completed completely and accurately, and that only one clinical study subject's data is recorded in each case report form. All data or text filled in incorrectly should not be altered, but should be drawn with a single line, and then a copy should be retained at the sponsor and site. Data entry requires an original CRF.

10. References

1. Sung H, *et al.* Global Cancer Statistics 2020: GLOBOCAN Estimates of Incidence and Mortality Worldwide for 36 Cancers in 185 Countries. *CA Cancer J Clin.* **71**, 209-249 (2021)
2. Cheon J, *et al.* Real-World Efficacy and Safety of Lenvatinib in Korean Patients with Advanced Hepatocellular Carcinoma: A Multicenter Retrospective Analysis. *Liver Cancer.* **9**, 613-624 (2020)
3. Li C, *et al.* Preoperative transcatheter arterial chemoembolization for surgical resection of huge hepatocellular carcinoma (≥ 10 cm): a multicenter propensity matching analysis. *Hepatol Int.* **13**, 736-747 (2019)
4. Wakayama K, *et al.* Huge hepatocellular carcinoma greater than 10 cm in diameter worsens prognosis by causing distant recurrence after curative resection. *J Surg Oncol.* **115**, 324-329 (2017)
5. Pawlik TM, *et al.* Tumor size predicts vascular invasion and histologic grade: Implications for selection of surgical treatment for hepatocellular carcinoma. *Liver Transpl.* **11**, 1086-1092 (2005)
6. Miyayama S, *et al.* Outcomes of conventional transarterial chemoembolization for hepatocellular carcinoma ≥10 cm. *Hepatol Res.* **49**, 787-798 (2019)
7. Vesselle G, *et al.* Predictive factors for complete response of chemoembolization with drug-eluting beads (DEB-TACE) for hepatocellular carcinoma. *Eur Radiol.* **26**, 1640-1648 (2016)
8. Song MJ, *et al.* Comparative study between doxorubicin-eluting beads and conventional transarterial chemoembolization for treatment of hepatocellular carcinoma. *J Hepatol.* **57**, 1244-1250 (2012)
9. Zhang ZS, Li HZ, Ma C, Xiao YD. Conventional versus drug-eluting beads chemoembolization for infiltrative hepatocellular carcinoma: a comparison of efficacy and safety. *BMC Cancer.* **19**, 1162 (2019)
10. Kudo M, *et al.* Randomised, multicentre prospective trial of transarterial chemoembolisation (TACE) plus sorafenib as compared with TACE alone in patients with hepatocellular carcinoma: TACTICS trial. *Gut.* **69**, 1492-1501 (2020)
11. Yang C, Qin S. Apatinib targets both tumor and endothelial cells in hepatocellular carcinoma. *Cancer Med.* **7**, 4570-4583 (2018)
12. Zhang XH, Cao MQ, Li XX, Zhang T. Apatinib as an alternative therapy for advanced hepatocellular carcinoma. *World J Hepatol.* **12**, 766-774 (2020)
13. Xue JM, *et al.* Efficacy and safety of apatinib treatment for gastric cancer, hepatocellular carcinoma and non-small cell lung cancer: a meta-analysis. *Onco Targets Ther.* **11**, 6119-6128 (2018)
14. Scott LJ. Apatinib: A Review in Advanced Gastric Cancer and Other Advanced Cancers. *Drugs.* **78**, 747-758 (2018)
15. Kan X, *et al.* Transarterial Chemoembolization Combined With Apatinib for Advanced Hepatocellular Carcinoma: A Propensity Score Matching Analysis. *Front Oncol.* **10**, 970 (2020)
16. Lu W, *et al.* Comparison of efficacy between TACE combined with apatinib and TACE alone in the treatment of intermediate and advanced hepatocellular carcinoma: A single-center randomized controlled trial. *Cancer Biol Ther.* **18**, 433-438 (2017)
17. Gu H, *et al.* Efficacy and safety of apatinib combined with transarterial chemoembolization (TACE) in treating patients with recurrent hepatocellular carcinoma. *Ann Transl Med.* **8**, 1677 (2020)
18. Hu Y, Hao M, Chen Q, Chen Z, Lin H. Comparison of the efficacy and safety among apatinib plus drug-eluting bead transarterial chemoembolization (TACE), apatinib plus

conventional TACE and apatinib alone in advanced intrahepatic cholangiocarcinoma. *Am J Transl Res*. **12**, 6584-6598 (2020)
19. Liu J*, et al.* Safety and Efficacy of Drug-Eluting Bead Transarterial Chemoembolization Combined with Apatinib in Patients with Advanced Hepatocellular Carcinoma. *Acad Radiol*. **27**, 704-709 (2020)
